# Harnessing microbial wealth for lignocellulose biomass valorization through secretomics: a review

**DOI:** 10.1186/s13068-021-02006-9

**Published:** 2021-07-05

**Authors:** Sivasamy Sethupathy, Gabriel Murillo Morales, Yixuan Li, Yongli Wang, Jianxiong Jiang, Jianzhong Sun, Daochen Zhu

**Affiliations:** School of the Environment and Safety Engineering, Biofuels Institute, Jiangsu University, Zhenjiang, 212013 Jiangsu China

**Keywords:** Depolymerization, Lignocelluloses, Proteomics, Secretomics, Metasecretomics, Glycoside hydrolases, Enzyme cocktails

## Abstract

The recalcitrance of lignocellulosic biomass is a major constraint to its high-value use at industrial scale. In nature, microbes play a crucial role in biomass degradation, nutrient recycling and ecosystem functioning. Therefore, the use of microbes is an attractive way to transform biomass to produce clean energy and high-value compounds. The microbial degradation of lignocelluloses is a complex process which is dependent upon multiple secreted enzymes and their synergistic activities. The availability of the cutting edge proteomics and highly sensitive mass spectrometry tools make possible for researchers to probe the secretome of microbes and microbial consortia grown on different lignocelluloses for the identification of hydrolytic enzymes of industrial interest and their substrate-dependent expression. This review summarizes the role of secretomics in identifying enzymes involved in lignocelluloses deconstruction, the development of enzyme cocktails and the construction of synthetic microbial consortia for biomass valorization, providing our perspectives to address the current challenges.

## Background

Lignocellulosic biomass is a gift of nature and its seen as an inexhaustible source of organic carbon for the green synthesis of diverse and biofuels and other bio-based compounds with very low (or null) carbon and sulphur emissions. The emergence of biomass-based industries in developed and developing countries has provided opportunities in diverse types of employment, as the use of biomass involves a series of logistics processes such as bulk collection, transportation and pretreatment [1].

Nearly 94% of the global lignocellulosic production comes from agricultural sources and represents ~ 140 gigatons annually [2], and is expected to further increase in response due to population growth, food demand and intensive agriculture. In addition, lignocellulosic biomass provides more environmental benefits than fossil-derived fuels and other processed compounds due to its renewable nature, high abundance and its capacity to degrade by microorganisms. Hence lignocellulosic biomass is recognized as an abundant and economic feedstock for the synthesis of valuable chemicals. However, most of the valuable biomass has being disposed by burning and landfilling in agricultural/domestic environments, or burned for electric power generation in several industries. In recent years, physical, thermochemical and biochemical approaches have been used as biomass pretreatment methods to produce syngas, fuel gas, bio-oil, biochar and bioethanol and other compounds. Though chemical and thermochemical processing methods are robust and efficient, biochemical methods such as bacterial, fungal and lignocellulolytic bacterial/fungal secretome-based pretreatment are becoming more relevant in recent years because of their eco-friendly nature and controlled depolymerization of biomass.

According to the global energy assessment (GEA), the energy derived from renewable resources is expected to cover 10–40% of the global energy requirements by 2050 [3, 4]. Typically, lignocellulosic biomass comprised cellulose (40–50%), hemicellulose (25–30%), lignin (3–30%), proteins, resins and others depending on the plant species (pectin, fatty acids, amino acids, pigments and secondary metabolites) [5, 6]. Cellulose is a linear polysaccharide consisting of glucose repeats linked by β − 1 → 4 glycosidic bonds. Whereas hemicellulose/polyose is a heteropolysaccharide made up of several different monosaccharides and its depolymerization is far easier than cellulose. Lignin is an aromatic heteropolymer composed of main three monolignols [coumaryl (H), coniferyl (G), and sinapyl alcohol (S)] and are cross-linked especially through β-O-4, 5-5, β-5, β-1, α-O-4, 4-O-5 and β–β bonds. In addition, O–H, C–H, C–O, C=C, and C–C bonds are also present in lignin [7, 8]. Since lignin is considered as the main source of aromatic compounds from renewable resources, it has attracted the attention of many researchers to produce a wide variety of value-added chemicals.

The massive use of lignocellulosic biomass still faces a series of challenges, some of them are the structural complexity of lignocellulosic biomass, the high lignin content, cellulose coverage by hemicellulose and lignin, cellulose’s crystalline nature, and the propensity of lignin to repolymerize. In the particular case of cellulose valorization, physical, chemical and biological pretreatment methods are being used to remove lignin and hemicellulose for its effective processing by microorganisms [9]. After lignocelluloses fractionation and cellulose conversion, in order to achieve a successful approach for lignin bioconversion to produce aromatic compounds at commercial scale, research has become more involved in the development of microbial tools. If the available biomass is used efficiently to produce biofuels and other several bio-based compounds, it would be possible to reduce fossil fuel consumption, to promote the circular economy and to decrease the global environmental burdens in the future. 

**Fig. 1:**
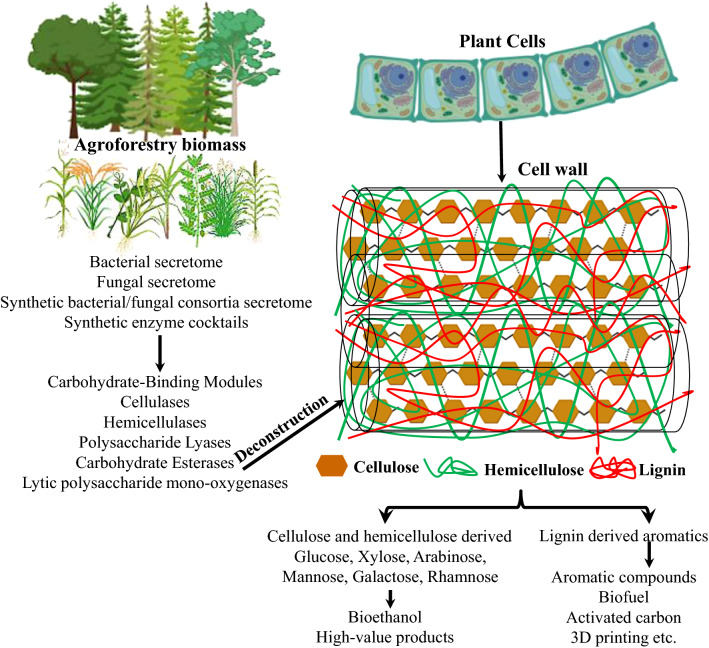
An overview of biomass degradation

Under natural circumstances, bacteria and fungi are actively involved in biomass deconstruction by hydrolysis, as these microorganisms secrete a series of hydrolytic enzymes, such as cellulases, hemicellulases, laccases, peroxidases, etc. [10] (Fig. 1). As a result, bacteria and fungi are used to transform lignocellulosic biomass into fermentable sugars and aliphatic/aromatic monomers. Microorganisms employ various mechanisms and secretory enzymes for deconstructing lignocellulosic biomass. However, the identification and purification of ligninolytic enzymes is a time-consuming process. Secretion of enzymes by lignocellulose degrading microbes depends on the nature and macromolecular composition of the biomass. In this regard, profiling of the bacterial/fungal secretomes using proteomic analyses is a befitting way to study the expression kinetics and identification of substrate-specific enzymes [11]. Numerous secretome analyses have catalogued the presence of active ligninolytic enzymes and their substrate-dependent expression pattern. Consequently, substrate-specific synthetic enzyme cocktails are developed and successfully used at laboratory scale as well as at industrial scale for biomass conversion.

To date, a number of synthetic enzyme cocktails and cellulase preparations are commercially available, for example, Accellerase 1000, Accellerase^®^ 1500, Novozyme 188, Celluclast 1.5 L, Cellic Ctec2, GC220, Depol ™ 692L, Spezyme-CP, etc. [11]. Microbial consortia are more efficient biomass degraders than single strains, due to the secretion of a repertoire of hydrolytic enzymes. However, sugars produced from biomass fermentation by microbial consortia treatment are rapidly utilized by some of the bacterial species present in the same microbial consortia, hampering their separation and use. Therefore, several studies demonstrated a significant increase in biofuel production from secretome/metasecretome pretreated biomass. Given the application of secretomes, metasecretomes and synthetic enzyme cocktails in biomass deconstruction/pretreatment for an environmentally friendly production of high-value products, this review focuses on the evaluation of the application of secretomic and metasecretomic technologies in the mining of enzymes associated with lignocelluloses fractionation and valorization.

### Glycoside hydrolases (GH)

Lignocellulosic degradation is a complex and multi-enzymatic process carried out predominantly by bacterial and fungal communities under natural conditions. Though fungi have been studied extensively for their application in lignocellulosic deconstruction, bacteria have attracted more attention owing to their genome size and suitability for genetic engineering and heterologous expression of desired enzymes/proteins and the expression of synthetic metabolic pathways [12]. Carbohydrate-active enzymes (CAZymes) are classified into four classes, namely glycoside hydrolases/glycosidases (GHs [EC 3.2.1.]), glycosyltransferases (GTs [EC 2.4.x.y]), polysaccharide lyases (PLs [EC 4.2.2.-]), carbohydrate esterases (CEs) and auxiliary activities (AAs). Nearly all life forms on Earth contain glycoside hydrolases (GHs) and can hydrolyse O-, N- and S-linked glycosidic linkages. Numerous investigations have reported the presence of GHs in bacteria, fungi, plants and animals. In particular, terrestrial fungi produce a plethora of extracellular and intracellular GHs to acquire nutrients from biomass [13–15]. GHs acts on glycosidic bonds in crystalline polysaccharides, amino polysaccharides and other complex polysaccharides present in the biomass and transform them into fermentable sugars [16]. Moreover, GHs are also associated with developmental processes, such as the defence against pathogens, basic cellular functions in higher eukaryotes [17, 18], parasitism in nematodes [19] and virulence in pathogenic fungi and bacteria [20]. It is interesting to note that GHs have been shown to participate in the inhibition of biofilm formation and the disruption of preformed biofilms by clinically identified bacterial pathogens [21–23]. Beta-galactosidase, amylases, cellulases and hemicellulases are the well-studied GHs and are used in a number of important biotechnological processes.

GHs are classified based on the location (intracellular and extracellular), the stereochemistry of hydrolysis (retaining and inverting) and the cleavage position (exo and endo) in the polysaccharide chain [24]. Likewise, based on the amino acid sequences and folding similarities, GHs are classified into 171 GH families. Among them, the GH families GH 5, GH13, GH16, GH30 and GH 43 are further classified into 56, 43, 27, 9 and 37 different sub-families, respectively [24, 67]. In addition, some of the GH families were classified under 18 clans (GH-A to GH-R) depending on the conserved folding patterns [25]. Based on the catalytic mechanisms, GHs are classified under the Enzyme Commission (EC) number. So far, terrestrial fungi have been reported to produce copious GHs which can degrade the complex lignocellulosic biomass. A recent study by Chrismas and Cunliffe (2020) have shown the presence of numerous GHs in the open ocean mycoplankton communities and the members of GH7 family was found to be the most abundant, followed by GH17, GH5, GH72, GH16, GH13, GH3, GH47, GH18, etc. [26]. Besides biomass pretreatment, GHs are mainly used in food, pharmaceutical, and paper and pulp industries. Despite the identification of numerous GHs, only a few of them are widely produced on a large scale for industrial applications due to their stability and requirement of stringent reaction conditions.

### Role of auxiliary activity (AA) lytic polysaccharide monooxygenases (LPMOs) in biomass hydrolysis

Numerous reports have demonstrated that the copper-containing lytic polysaccharide monooxygenases (LPMOs) are mainly present in fungal and bacterial kingdoms. Most of them have been identified over the past decade and have shown to improve the degradation of lignocellulosic biomass through oxidative cleavage of glycosidic linkages [27, 28]. For the first time, a secreted LMPO called catalytic chitin-binding protein (CBP21) by *Serratia marcescens* was shown to enhance the chitin degradation by binding with chitin and inducing structural changes, which makes chitin more accessible for chitinases [29].

According to the information available at the Carbohydrate-Active enZymes (CAZy) database, to date, the identified auxiliary activity (AA) enzymes/proteins are classified into 16 families, among them 6 families such as AA9, AA10, AA11, AA13, AA14 and AA15, consisted of LPMOs [30]. The most common AA enzyme in fungi is AA9 which acts on cellulose, while AA13 and AA14 act on chitin, starch [31] and xylan [32], respectively. Chitin-active LPMOs are classified under the AA10, AA11, and AA15 families and are present in bacteria, fungi, viruses and arthropods [33]. A new AA16 family identified in *Aspergillus aculeatus* was shown to increase the activity of *T. reesei* cellobiohydrolase I by increasing the substrate availability [34]. The prevalence of LPMOs in the bacterial and fungal population involved in the lignocellulosic biomass degradation highlight the important role of LPMOs in the recycling of organic carbon in nature [35]. However, LPMOs are also associated with the virulence in phytopathogenic bacteria/fungi as well as in some human pathogenic *Vibrio cholerae* and *Listeria monocytogenes* [36, 37]. LPMOs are used as a key component in the widely used cellulase and hemicellulase cocktail called Cellic CTec enzyme products (Novozymes A/S) at industrial scale for ethanol production using lignocellulosic biomass [35].

The presence of β-sandwich folds in the core structure and a flat substrate binding site in LPMOs provides the much-needed flexibility to act on the crystalline cellulose and other polymers [38, 39]. Fungal LPMOs contain histidine brace with a copper ion and *N*-methylated N-terminal histidine (His1) [40, 41], whereas bacterial LPMOs contains only histidine brace with copper in their active sites [39, 42]. The catalytic activity of LPMOs depends on the reduction of copper followed by oxidative cleavage of β−(1 → 4) glycosidic linkage and the oxidation of the C1 or C4 position of the substrate in the presence of O_2_ as a co-substrate [27, 43–45]. Studies by Müller et al., [46] and Bissaro et al. [47] suggested that H_2_O_2_ can be used as a co-substrate by LPMOs and the supplementation of H_2_O_2_ was found to enhance the saccharification of cellulose by the Cellulase cocktail Cellic^®^ CTec2 containing LPMOs.

### Occurrence of AA LPMOs in bacteria and fungi

According to the CAZy database, it is apparent that the AA10 family (6938 entries-accessed on 15th June 2020) is very common in bacteria. However, only a small fraction of AA10 LPMOs has been experimentally well studied and validated. For example, AA10 LPMOs from *Bacillus amyloliquefaciens* DSM7, *Bacillus licheniformis*, *Bacillus thuringensis*, *Caldibacillus cellulovorans*, *Cellvibrio japonicus* Ueda107, *Enterococcus faecalis* V583, *Hahella chejuensis* KCTC 2396, *Jonesia denitrificans* DSM20603, *Listeria monocytogenes* EGD-e, *Photorhabdus laumondii* subsp. laumondii TTO1, *Serratia marcescens* BJL200, *Streptomyces ambofaciens* ATCC 23877, *Streptomyces coelicolor* A3(2), *Streptomyces griseus*, *Streptomyces lividans* 1326, *Streptomyces pratensis* ATCC 33331, *Teredinibacter turnerae* T7901, *Thermobifida fusca* YX and *Vibrio cholerae* O1 biovar El were characterized. Recently identified LPMO from *Photorhabdus luminescen* called Pl AA10 can act on both cellulose and chitin [48].

Furthermore, members of *Ascomycetes* such as *Neurospora crassa* [49, 50]*, Pestalotiopsis* sp. [51], *Pestalotiopsis *sp NCi6 [52], *Trichoderma asperellum* [53]*, Fusarium graminearum* [54, 55]*, Fusarium verticillioides* [56]*, Penicillium oxalicum* [57]*, Aspergillus niger* [58]*, Aspergillus nidulans* [59, 60]*, Aspergillus fumigatus* [31], *Myceliophthora thermophila* [61], *Penicillium echinulatum* [62]*, Malbranchea Cinnamomea* [63] and *Podospora anserina* [64], and members of Basidiomycetes such as *Gloeophyllum trabeum* [65, 66]*, Laetisaria arvalis* [67]*, Phanerochaete chrysosporium* [68, 69]*, Pycnoporus coccineus* [70]*, Pycnoporus sanguineus, Pycnoporus cinnabarinus* [71] and *Schizophyllum communae* [72] were reported to have AA enzyme genes and their expression is dependent on biomass nature. In addition, *Myceliophthora thermophila* [73, 74]*, Thielavia terrestris* [75]*, Geotrichum candidum* [76], *Heterobasidion irregulare* [77], *Thermoascus aurantiacus* [41, 78] were also shown to have LMPOs of biotechnological importance. However, experimental validation is required for the addition of one or more appropriate AAs into commercially available enzyme cocktails to expand their application in the degradation of various biomass resources.

### Ligninolytic enzymes

Lignin removal is an important and difficult process in biomass conversion as it acts as a protective barrier to cellulose. Chemical degradation of lignin is an energy-intensive process which requires extreme reaction conditions (high temperature, high pressure and acidic/alkaline pH). Microbes break down lignin aerobically and anaerobically and are considered as the best choice for lignin degradation. Furthermore, ligninolytic microbes are useful in the synthesis of aromatic compounds of interest of the food flavouring and pharmaceutical industries [79, 80]. The ligninolytic system of bacteria actinomycetes and fungi is mainly comprised laccases (LACs [EC 1.10.3.2]), versatile peroxidases (VP [EC 1.11.1.16]), lignin peroxidases (LiPs [EC 1.11.1.14]) and manganese peroxidases (MnPs [EC 1.11.1.13]) [79–81]. Although ligninolytic enzymes were first identified from fungus, it was later found that the ligninolytic enzymes from bacterial sources are more effective in the deconstruction of various types of lignin such as kraft, alkali, organosolv, soda and sulfolignins. Among the ligninolytic enzymes, LACs find a place in the alcohol and food industries in the improvement of wine, beer, fruit juice, bread and sugar beet pectin’s quality [82–84]. LACs are muliti-copper oxidoreductases (MCOs), ubiquitously present in bacteria and fungi, and have been shown to deconstruct lignin and polyaromatic hydrocarbons, dye discolouration as well [85–87]. The availability of the microbial whole genome and whole metagenomic data paved the way for exploring the presence of lignin deconstructing enzymes using bioinformatic tools.

Ausec et al., reported more than 1200 putative MCOs in 2,200 whole and draft bacterial genomes, and four metagenomic sequence sets. Among the identified putative MCOs encoding genes, 76% of the genes are predicted to have a signal peptide indicating that the majority of MCOs are secreted into the environment to degrade lignin [88]. Genomic reconstruction of lignin degradation pathways in *Cupriavidus basilensis* B-8 revealed the presence of three functional pathways [89]. Briefly, β-ketoadipate pathway with the catechol branch and the protocatechuate branch, phenol degradation pathway and gentisate pathway for the catabolism of lignin and lignin-derived aromatics. Further analysis showed that manganese peroxidase (MnP) is required for the growth of *C. basilensis* in the presence of lignin and in a subsequent stage of growth laccase has been found to degrade lignin [89]. In a recent functional genomic study, 13 bacterial genomes were examined to identify pathways involved in lignin deconstruction. The results showed that lignin degradation is a complex process involving DyP-type peroxidases and MCOs, aromatic compounds degrading gene clusters and the β-ketoadipate pathway [90].

### What is the need for proteomic analysis of lignocellulolytic microorganisms?

Under different growth conditions (pH, temperature and media composition/biomass) lignocellulose-hydrolysing bacteria and fungi secret different types of GHs (Table 1). Moreover, studies have shown the difference in the secretion and proportion of GHs by lignocellulolytic microbes when they have grown in solid state, static and in submerged fermentation modes (Table 1). Thus, it is important to analyse the secretome of lignocellulolytic strains grown on specific biomass to study the substrate-dependent secretion pattern of GHs, identification of novel and potent GHs for biomass hydrolysis. Genomic analysis of *T. reesei* has revealed the presence of genes encoding extracellular cellobiohydrolases (*N* = 2), extracellular endoglucanases (*N* = 8), xylanases (extracellular, *N* = 4 and intracellular, *N* = 1), intracellular xylanase (*N* = 1), glycosidases (extracellular, *N* = 5, intracellular, *N* = 6 and mitochondrion, *N* = 1) and xylosidases (extracellular, *N* = 1 and intracellular, *N* = 1) [91]. Nevertheless, proteomic analysis of secretomes of *T. reesei* QM6a and Rut C30 have grown on different lignocellulosic biomass revealed that the expression of lignocellulolytic enzymes are modulated by the nature and complex composition of the biomass to be degraded [92–94]. For example, enzymes such as endoglucanases (*N* = 18), glucosidases (*N* = 13), xylanases, arabinofuranosidases, β-xylosidases, α- and β-glucuronidases, CBM1 acetylxylan esterase, CBM1 acetyl esterase, cellobiohydrolase, β-mannase, α- and β-mannosidases, polysaccharide lyases, exo-rhamnogalacturonase, endopolygalacturonase, 3-phytase, and glucuronan lyase A were secreted by *T. reesei* (QM6a and Rut C30) when cultured on fibrous cellulose, corn stover, and sawdust (1% W/V) [92]. In addition, proteomic analysis highlighted the expression of substrate binding proteins such as cellulose-binding domain (CBM1) Cip2, carbohydrate-binding module family 13 and hydrophobin-1 and 2 [92]. Furthermore, enzymes involved in lignin degradation such as laccase, superoxide dismutase, glyoxal oxidase, peroxidases and oxidoreductases were identified and quantified from the secretome of *T. reesei* QM6a and Rut C30. Importantly, proteomic analysis leads to a better understanding of the importance of auxiliary proteins in degrading lignocellulosic biomass [92]. Global proteomic analysis of *T. reesei* QM9414 cultured on glucose, cellulose and sophorose as carbon sources revealed the differential regulation of several genes encoding accessory proteins, transcriptional regulators, transporters, major facilitator superfamily (MFS) permeases and CAZymes. Hierarchical clustering and gene regulatory network analyses demonstrated that 75 and 107 genes were specific to sophorose and cellulose utilization, respectively [95].

**Table 1:** List of lignocellulosic biomass-degrading microbe’s secretome analysed

Fungi	Substrate and culture conditions	Proteomic analysis	Distribution of GHs in the secretome of lignocellulolytic fungi	References
*Doratomyces stemonitis* C8	Avicel cellulose (1.5%), 250 rpm, 7 days, 28 °C	2D-PAGE, LC–MS/MS	GH3 (*N* = 4), GH5 (*N* = 4), GH7 (*N* = 3), GH10 (*N* = 2), GH43 (*N* = 2), GH62, GH74, GH78	[206]
*A. fumigatus* AF293	Sugarcane bagasse (1%), 37 °C, 24 h	SDS-PAGE LC–MS/MS	GH1, GH2, GH3, GH6, GH7 (*N* = 3), GH10 (*N* = 2), GH11 (*N* = 2), GH13, GH131, GH15, GH16 (*N* = 5), GH17 (*N* = 2), GH18 (*N* = 4), GH25, GH27(*N* = 2), GH28, GH30, GH43 (*N* = 6), GH47 (*N* = 2), GH5 (*N* = 7), GH53, GH55, GH62 (*N*-2), GH65, GH71, GH72 (*N* = 2), GH76, GH81, GH92, GH93	[154]
*A. fumigatus* Z5	Rice straw (1%), 50 °C, 170 rpm, 7 days	iTRAQ LC–MS/MS	GH3 (*N* = 2), GH5, GH6, GH7 (*N* = 2), GH10 (*N* = 3), GH11 (*N* = 2), GH12, GH15, GH16, GH17, GH18 (*N* = 4), GH27, GH30, GH43 (*N* = 3), GH47, GH61(*N* = 2), GH62	[152]
*Fusarium verticillioides*	Wheat straw, 30 °C, 110 rpm 7 days	1D-PAGE LC–MS/MS	GH1, GH3 (*N* = 6), GH5 (*N* = 2), GH7, GH10 (*N* = 4), GH11, GH13, GH15 (*N* = 2), GH16, GH27 (*N* = 3), GH28 (*N* = 2), GH29 (*N* = 2), GH30, GH31, GH32, GH39, GH43 (*N* = 8), GH51, GH54, GH61 (*N* = 2), GH62, GH67, GH76, GH79, GH88, GH93 (*N* = 2)	[207]
*T. reesei* Rut-C30	Sugarcane bagasse (1%), 30 °C, 200 rpm, 72 h	LC–MS/MS	GH1 (*N* = 2), GH2 (*N* = 2), GH3 (*N* = 2), GH5 (*N* = 3), GH6, GH7, GH10 (*N* = 2), GH11(*N* = 3), GH12, GH15, GH16 (*N* = 3), GH17 (*N* = 3), GH18 (*N* = 2),GH20, GH28 (*N* = 2), GH31, GH43 (*N* = 5), GH47 (*N* = 2), GH55, GH57, GH61(*N* = 2), GH67, GH71(*N* = 4), GH72 (*N* = 2), GH74 (*N* = 2), GH75, GH92 (*N* = 2)	[208]
*T. reesei* Rut-C30	Sugarcane bagasse (1%), 30 °C, static for 24 h, 30 °C, 200 rpm, 48 h	LC–MS/MS	GH2, GH3, GH5, GH6, GH7, GH10 (*N* = 2), GH11(*N* = 4), GH12, GH15, GH16, GH18 (*N* = 2), GH31, GH38, GH47 (*N* = 2), GH57, GH61, GH67 (*N* = 2), GH71 (*N* = 2), GH74, GH75, GH92	[208]
*T. reesei* Rut-C30	Sugarcane bagasse, 0 °C, 3 days	1D-PAGE LC–MS/MS	GH1 (*N* = 2), GH2, GH3 (*N* = 5), GH5 (*N* = 2), GH6 (*N* = 2), GH7 (*N* = 2), GH11 (*N* = 3), GH20, GH25, GH27 (*N* = 2), GH28, GH30, GH31, GH35, GH36,GH37, GH54 (*N* = 2), GH55, GH61 (*N* = 2), GH62, GH67, GH72 (*N* = 2), GH74, GH92	[53]
*Trichoderma asperellum* S4F8	Sugarcane bagasse, 30 °C, 3 days	1D-PAGE LC–MS/MS	GH1 (*N* = 2), GH2, GH3 (*N* = 6), GH5 (*N* = 5), GH6, GH7 (*N* = 2), GH10, GH11(*N* = 4), GH12, GH15 (*N* = 2), GH16 (*N* = 2), GH17, GH18 (*N* = 3), GH20, GH25, GH27 (*N* = 2), GH28 (*N* = 2), GH31 (*N* = 2), GH35, GH36, GH43, GH47, GH54 (*N* = 2), GH55, GH62 (*N* = 2), GH67, GH72 (*N* = 3), GH74, GH79, GH92 (*N* = 3), GH93, GH95	[53]
*A. niger* A12	Sugarcane bagasse (1%), 32 °C, 200 rpm, 72 h	LC–MS/MS	GH2, GH3 (*N* = 2), GH5, GH7, GH11(*N* = 2), GH12, GH15, GH16, GH18, GH20, GH31 (*N* = 2), GH32, GH35, GH38 (*N* = 2), GH43, GH47, GH62 (*N* = 2), GH64, GH72 (*N* = 2)	[208]
*A. niger* A12	Sugarcane bagasse (1%),, 32 °C, static for 24 h, 32 °C, 200 rpm, 48 h	LC–MS/MS	GH2 (*N* = 2), GH3 (*N* = 4), GH5, GH7, GH11(*N* = 3), GH12(*N* = 3), GH15, GH16, GH31 (*N* = 2), GH32, GH35, GH43 (*N* = 2), GH47, GH62 (*N* = 2), GH64, GH72 (*N* = 3)	[208]
*A. nidulans* strain FGSCA4	Wheat starch (1%), 30 °C, 150 rpm, 5 days	LC–MS/MS	GH1, GH2, GH3 (*N* = 5), GH5, GH6, GH7, GH10, GH11, GH13 (*N* = 13), GH15, GH16 (*N* = 6), GH17 (*N* = 2), GH20 (*N* = 2), GH25 (*N* = 3),GH27 (*N* = 2), GH28, GH31 (*N* = 3), GH36, GH43 (*N* = 4), GH47, GH53, GH54, GH55 (*N* = 2),GH62, GH63, GH65, GH71 (*N* = 2), GH72 (*N* = 3), GH74, GH76, GH81, GH92 (*N* = 3), GH93, GH105, GH125, GH132 (*N* = 2)	[59]
*A. nidulans* strain FGSC A4	High-amylose maize starch (1%), 30 °C, 150 rpm, 5 day	LC–MS/MS	GH1, GH2, GH3 (*N* = 5), GH5 (*N* = 3), GH6, GH7, GH10, GH11, GH13 (*N* = 13), GH15, GH16 *N* = (6), GH17 (*N* = 2), GH20 (*N* = 2), GH24, GH25 (*N* = 3), GH27 (*N* = 2), GH28 (*N* = 2), GH31 (*N* = 3), GH35, GH36, GH43 (*N* = 4), GH47, GH53, GH54, GH55 (*N* = 2), GH62, GH63, GH65, GH71 (*N* = 2), GH72 (*N* = 3), GH74, GH76, GH81, GH92 (*N* = 3), GH93,GH105, GH125, GH132 (*N* = 2)	[59]
*A. nidulans* strain FGSC A4	Pea starch (1%), 30 °C, 150 rpm, 5 day	LC–MS/MS	GH1, GH2, GH3 (*N* = 5), GH6, GH10, GH11, GH13 (*N* = 13), GH15, GH16 (*N* = 6), GH17 (*N* = 3), GH20 (*N* = 2), GH25 (*N* = 3), GH27 (*N* = 2), GH28, GH31 (*N* = 3), GH35, GH36, GH43 (*N* = 4), GH47, GH54, GH55 (*N* = 2), GH63, GH65, GH71 (*N* = 2), GH72 (*N* = 3), GH74, GH76, GH81, GH92 (*N* = 3), GH93, GH95, GH105, GH125, GH132 (*N* = 2)	[59]
*Aspergillus fumigatus* AF14	Corn straw:wheat bran (1:1), solid-state fermentation	LCMS/MS	GH1, GH2, GH3 (*N* = 7), GH5 (*N* = 5), GH6, GH7 (*N* = 5), GH10 (*N* = 3), GH11, GH12 (*N* = 2), GH17, GH27 (*N* = 3), GH35 (*N* = 3), GH38, GH43, GH47, GH51, GH54, GH61, GH67	[209]
*Phanerochaete chrysosporium*	Corn stover (3 g/L), static, 28 °C	1D-PAGE LC–MS/MS	GH1, GH2 (*N* = 2), GH3 (*N* = 6), Gh5 (*N* = 7), GH6, GH7(*N* = 4), GH10 (*N* = 4), GH12, GH13 (*N* = 3), GH-15, GH16 (*N* = 6), GH18 (*N* = 6), GH20 (*N* = 4), GH28 (*N* = 3), GH30 (*N* = 2), GH31 (*N* = 6), GH35 (*N* = 2), GH37, GH38, GH43, GH47, GH51, GH55 (*N* = 2), GH72, GH74, GH78, GH79 (*N* = 2), GH88, GH89, GH92 (*N* = 3), GH95, GH115, GH125, GH131 (*N* = 2), GH133	[69]
*P. chrysosporium*	Corn stover (1 g/L), 28 °C, 150 rpm	1D-PAGE LC–MS/MS	GH1, GH2 (*N* = 2), GH3 (*N* = 5), GH5 (*N* = 5), GH6, GH7, GH10 (*N* = 3), GH13 (*N* = 4), GH15. GH16 (*N* = 2), GH18 (5), GH20, GH28 (*N* = 3), GH30, GH31 (*N* = 4), GH35, GH47. GH63, GH72, GH74 (*N* = 4), GH92 (*N* = 2), GH95, GH125, GH131	[69]
*P. chrysosporium* strain K-3	Ammonia-treated birch wood meal (2%)	2D-PAGE LC/MS–MS	GH3, GH5 (*N* = 3), GH6 (*N* = 2), GH7 (*N* = 8), GH10 (*N* = 7), GH11 (*N* = 2), GH12 (*N* = 2), GH28 (*N* = 2), GH43, GH45, GH72 (*N* = 4)	[79]
*P. chrysosporium*	Cellulose (1%), 30 °C and 100 rpm, 14 days	1D-PAGE, iTRAQ LC–MS/MS	GH1 (*N* = 2), GH2 (*N* = 2), GH3 (*N* = 2), GH4, GH5 (*N* = 9), GH6, GH7 (*N* = 3), GH10 (*N* = 2). GH11, GH12, GH13 (*N* = 3), GH15, GH16 (*N* = 2), GH17 (*N* = 2), GH18 (*N* = 3), GH20, GH27, GH28 (*N* = 2), GH31, GH35, GH43, GH47 (*N* = 2), GH61, GH72, GH74, GH79, GH95	[210]
*P. chrysogenum* P33	Combination of different biomasses (1%), 30 °C, 200 rpm, 36 h	1D-PAGE LC–MS/MS	GH1 (*N* = 3), GH2, GH3 (*N* = 4), GH5, GH6, GH7 (*N* = 2), GH10 (*N* = 2), GH11, GH12 (*N* = 3), GH27, GH35, GH36, GH43 (*N* = 6), GH51, GH54, GH62, GH76, GH127	[211]
*Penicillium* *funiculosum* NCIM 1228	wheat bran (2.4%) and Avicel cellulose (2.14%), 30 °C for 6 days, 150 rpm	1D-PAGE, LC–MS/MS	GH1, GH2, GH3 (*N* = 12), GH5 (*N* = 3), GH7 (*N* = 2), GH6, GH10, GH11 (*N* = 5), GH12 (*N* = 2), GH13 (*N* = 2), GH15 (*N* = 4), GH16 (*N* = 5), GH17, GH18 (*N* = 4), GH20, GH27 (*N* = 2), GH28 (*N* = 2), GH30 (*N* = 3), GH31 (*N* = 4), GH35 (*N* = 3), GH43 (*N* = 3), GH51, GH53, GH54 (*N* = 2), GH-55, GH62 (*N* = 4), GH64 (*N* = 2), GH65, GH67, GH71 (*N* = 3), GH72 (*N* = 44), GH74, GH79, GH88, GH92 (*N* = 2), GH93, GH105	[109]
*Penicillium* sp. Dal 5	Cellulose (3%), wheat bran (1%) and rice straw (1%), 30 °C, 180 rpm, 7 days	1D-PAGE LC–MS/MS	GH2 (*N* = 3), GH3 (*N* = 4), GH5 (*N* = 4), GH6, GH7 (*N* = 3), GH10 (*N* = 2), GH12, GH15 (*N* = 2), GH16, GH17 (*N* = 2), GH18 (*N* = 3), GH20, GH25, GH28, GH31, GH35, GH43 (*N* = 4), GH47, GH61, GH62 (*N* = 2), GH64, GH65, GH72 (*N* = 3), GH81, GH92	[212]
*P. oxalicum* JU-A10	Commercial cellulase preparations (CCP)	LC–MS/MS	GH2 (*N* = 3), GH3 (*N* = 5), GH5 (*N* = 6), GH6, GH7 (*N* = 3), GH10 (*N* = 3), GH11 (*N* = 1), GH12 (*N* = 2), GH13, GH15 (*N* = 2), GH16 (*N* = 3), GH17 (*N* = 2), GH18 (*N* = 3), GH20, GH25, GH27, GH28 (*N* = 8), GH4 (*N* = 30), GH35. GH43 (*N* = 5), GH45, GH47, GH53, GH54, GH55, GH61 (*N* = 2), GH62 (*N* = 2), GH64, GH65, GH67, GH72 (*N* = 4), GH79, GH92, GH93	[213]
*P. echinulatum* strain 9A02S1-DSM18942	Integral sugar cane bagasse (1%), 29 °C, 200 rpm, 5 days	LC–MS/MS	GH3 (*N* = 2), GH5 (*N* = 6), GH6 (*N* = 4), GH7 (*N* = 10), GH12, GH61, GH17 (*N* = 2), GH10 (*N* = 3), GH11 (*N* = 2), GH62 (*N* = 2), GH43 (*N* = 2), GH28 (*N* = 2), GH18	[214]
	Hydrothermally treated bagasse (1%), 29 °C, 200 rpm, 5 days	LC–MS/MS	GH3, GH5 (*N* = 5), GH6 (*N* = 5), GH7 (*N* = 19), GH62, GH13	[214]
	Steam-explosion treated sugar cane bagasse (1%), 29 °C, 200 rpm, 5 days	LC–MS/MS	GH3, GH5 (*N* = 5), GH6 (*N* = 6), GH7 (*N* = 15), GH10 (*N* = 2), GH11	[214]
	Sulfuric acid treated Sugar cane bagasse (1%)	LC–MS/MS	GH3, GH5 (*N* = 5), GH6 (*N* = 6), GH7 (*N* = 15), GH17, GH10, GH18 (*N* = 2), GH65, GH92	[214]
	Microcrystalline cellulose (1%), 29 °C, 200 rpm, 5 days,	LC–MS/MS	GH3 (*N* = 2), GH5 (*N* = 4), GH6 (*N* = 4), GH7 (*N* = 11), GH12, GH17, GH18 (*N* = 4), GH20 (*N* = 2)	[214]
*Trichoderma reesei* SN1	Commercial cellulase preparation	LC–MS/MS	GH2 (*N* = 2), GH3 (*N* = 5), GH5 (*N* = 4), GH6, GH7 (*N* = 3), GH10, GH11, GH12, GH13, GH15, GH16 (*N* = 2), GH17 (*N* = 3), GH18 (*N* = 3), GH20, GH25, GH27 (*N* = 2), GH28, GH30, GH31, GH35 (*N* = 2), GH36, GH37, GH45, GH54 (*N* = 2), GH55 (*N* = 2), GH61 (*N* = 2), GH62, GH64, GH65, GH67, GH72 (*N* = 3), GH74, GH76, GH92	[213]
*Schizophyllum commune* SH12	Jerusalem artichoke stalk (3%), static, 28 °C, 5 days	1D-PAGE LC–MS/MS	GH1 (*N* = 2), GH2 (*N* = 2), GH3 (*N* = 5), GH5 (*N* = 4), GH6, GH7 (*N* = 2), GH10 (*N* = 3), GH11, GH13 (*N* = 5), GH15, GH16 (*N* = 4), GH17, GH18 (*N* = 7), GH20, GH27, GH28, GH30, GH31 (*N* = 4), GH32, GH35 (*N* = 5), GH37, GH38, GH43 (*N* = 10), GH45, GH47, GH51 (*N* = 2), GH53, GH55, GH62, GH71, GH72, GH74, GH76 (*N* = 5), GH81, GH88 (*N* = 3), GH92 (*N* = 3), GH93, GH115	[72]
*Phanerochaete chrysosporium* PC2	Jerusalem artichoke stalk (3%), static, 28 °C, 5 days	1D-PAGE LC–MS/MS	GH3 (*N* = 5), GH5 (*N* = 5), GH6, GH7 (*N* = 2), GH10 (*N* = 4), GH12, GH13 (*N* = 3), GH15, GH16 (*N* = 3), GH18 (*N* = 7), GH20, GH27, GH28, GH30, GH31, GH37,GH43, GH47, GH51, GH55, GH71, GH72, GH74, GH88 (*N* = 2), GH92 (*N* = 3), GH131	[72]
*Ceriporiopsis subvermispora* CBS 347.63	Jerusalem artichoke stalk (3%), static, 28 °C, 5 days	1D-PAGE LC–MS/MS	GH2, GH3 (*N* = 2), GH5 (*N* = 3), GH10 (*N* = 3), GH15, GH18, GH20, GH27, GH28 (*N* = 2), GH35, GH37,GH43, GH47, GH51, GH55, GH72, GH88, GH92 (*N* = 2), GH95	[72]
*Gloeophyllum trabeum*	Jerusalem artichoke stalk (3%), static, 28 °C, 5 days	1D-PAGE LC–MS/MS	GH1, GH3 (*N* = 3), GH5 (*N* = 5), GH10 (*N* = 3), GH12, GH13, GH15, GH16 (*N* = 3), GH18 (*N* = 7), GH20, GH27, GH28 (*N* = 5), GH29, GH30 (*N* = 2), GH31 (*N* = 3), GH35 (*N* = 2), GH37, GH43 (*N*-2), GH47, GH51 (*N* = 2), GH55, GH71, GH72, GH74, GH76 (*N* = 2), GH78, GH79, GH88, GH92 (*N* = 3), GH115	[72]
*Malbranchea cinnamomea* CM-10 T	Sorghum straw, static,45 °C, 7 days	1D-PAGE LC–MS/MS	GH1, GH2 (*N* = 3), GH3 (*N* = 3), GH5 (*N* = 2), GH6, GH7 (*N* = 2), GH10 (*N* = 3), GH18 (*N* = 2), GH20, GH35, GH47, GH55 (*N* = 2), GH67, GH81, GH92, GH125	[63]
*Thermobifida cellulosilytica* TB100T	Rice straw (1%), 50 °C, 150 rpm, 7 days	LC–MS/MS	GH5 (*N* = 2), GH6 (*N* = 2), GH9 (*N* = 2), GH10 (*N* = 2), GH43, GH48,GH81	[215]
*Cellulomonas fimi* ATCC 484	CMC (0.2%), 30 °C, 30 h	1D-PAGE LC–MS/MS	GH3 (*N* = 2), GH5 (*N* = 2), GH6 (*N* = 3), GH9 (*N* = 2), GH10 (*N* = 3), GH13 (*N* = 2), GH16, GH18, GH23, GH26, GH48, GH51, GH64, GH81, GH94	[216]
	Xylan (0.2%), 30 °C, 30 h	1D-PAGE, LC–MS/MS	GH3 (*N* = 4), GH5 (*N* = 2), GH6 (*N* = 3), GH9 (*N* = 2), GH10 (*N* = 2), (*N* = 3), GH11, GH13 (*N* = 2), GH16, GH18, GH39, GH43, GH51 (*N* = 2), GH62, GH64, GH81	[216]
	CMC (0.2%), 30 °C, 30 h	1D-PAGE, LC–MS/MS	GH6 (*N* = 3), GH5 (*N* = 2), GH9 (*N* = 3), GH10 (*N* = 8), GH11 (*N* = 3), GH13, GH26, GH27, GH43 (*N* = 2), GH48, GH62, GH68, GH81, GH92, GH105	[216]
	Xylan (0.2%), 30 °C, 30 h	1D-PAGE, LC–MS/MS	GH6 (*N* = 3), GH5 (*N* = 2), GH9 (*N* = 4), GH10 (*N* = 10), GH11 (*N* = 3), GH26, GH39 (*N*-2), GH43, GH51, GH62, GH92	[216]
*Penicillium echinulatum* 2HH	Steam-explosion treated sugar cane bagasse, 28 °C, 96 h	1D-PAGE, LC–MS/MS	GH3, GH5 (*N* = 5), GH6, GH7 (*N* = 2), GH10 (*N* = 2), GH12, GH13, GH30, GH43 (*N* = 2), GH47, GH99	[62]
*P. echinulatum* 2HH	Cellulose	1D-PAGE, LC–MS/MS	GH3 (*N* = 2), GH6, GH7 (*N* = 2), GH5 (*N* = 6), GH10 (*N* = 2), GH12, GH13, GH30, GH132, GH43 (*N* = 2), GH47, GH93, GH99, GH125	[63]
*Lentinula edodes*	Cellulose (2%), static 25 °C, 20 days	LC–MS/MS	GH2, GH3, GH5 (*N* = 2), GH6, GH7 (*N* = 2), GH10 (*N* = 2), GH12, GH15, GH27 (*N* = 2), GH28 (*N* = 4), GH35, GH43 (*N* = 2), GH51 (*N* = 2), GH53, GH74, GH95, GH115	[217]
*Pleurotus eryngii*	Ramie stalk, static, 28 °C, 21 days	1D-PAGE LC–MS/MS	GH1, GH2, GH3 (*N* = 2), GH3 (*N* = 3), GH7, GH15, GH16 (*N* = 6), GH17, GH18 (*N* = 2), GH20 (*N* = 2), GH27, GH31 (*N* = 5), GH35 (*N* = 2), GH38 (*N* = 2), GH45, GH51 (*N* = 2), GH55, GH63, GH71, GH79, GH92, GH95	[218]
*Phanerochaete chrysosporium*	Ramie stalk, static, 28 °C, 21 days	1D-PAGE LC–MS/MS	GH2, GH3, GH5 (*N* = 2), GH7 (*N* = 6), GH12 (*N* = 2), GH16 (*N* = 2), GH17, GH20, GH31 (*N* = 2), GH35, GH38, GH51 (*N* = 2), GH63, GH79, GH92, GH95	[218]
*Irpex Lacteus*	Ramie stalk, static, 28 °C, 21 days	1D-PAGE LC–MS/MS	GH1, GH2, GH3, GH5 (*N* = 5), GH7 (*N* = 4), GH13 (*N* = 2), GH16 (*N* = 6), GH18, GH20, GH27, GH31 (*N* = 4), GH35 (*N* = 3), GH38, GH45, GH51 (*N* = 2), GH63, GH71, GH79 (*N* = 2), GH92 (*N* = 4)	[218]
*Pleurotus ostreatus*	Ramie stalk, static, 28 °C, 21 days	1D-PAGE LC–MS/MS	GH1, GH2, GH3, GH5,GH7 (*N* = 4), GH15, GH16 (*N* = 4), GH18, GH20, GH27, GH31, GH35, GH45, GH51 (*N* = 2), GH63, GH71, GH79 (*N* = 3), GH92 (*N* = 2)	[218]
*Clonostachys byssicola*	Avicel cellulose (1%), 28 °C, 120 rpm, 7 days	LC–MS/MS	GH2 (*N* = 3), GH3 (*N* = 5), GH5 (*N* = 7), GH6 (*N* = 3), GH7 (*N* = 4), GH10 (*N* = 5), GH12, GH15, GH16 (*N* = 3), GH17, GH18, GH26 (*N* = 2), GH28 (*N* = 3), GH30 (*N* = 2), GH31 (*N* = 2), GH33, GH43 (*N* = 7), GH45, GH47, GH53, GH54 (*N* = 2), GH55, GH62, GH67, GH72 (*N* = 4), GH78, GH93 (*N* = 2), GH95, GH105, GH115, GH125, GH131	[219]
*C. byssicola*	CMC (1%), 28 °C, 120 rpm, 7 days	LC–MS/MS	GH2, GH3 (*N* = 2), GH5 (*N* = 6), GH6 (*N* = 2), GH7 (*N* = 4), GH10 (*N* = 4), GH11 (*N* = 2), GH15, GH16 (*N* = 2), GH17, GH26, GH30 (*N* = 2), GH31, GH47, GH53, GH55, GH67, GH72 (*N* = 2), GH115, GH131	[219]
*C. byssicola*	Oat spelt xylan (1%), 28 °C, 120 rpm, 7 days	LC–MS/MS	GH1, GH2 (*N* = 2), GH3 (*N* = 3), GH5 (*N* = 3), GH7, GH10 (*N* = 4), GH11 (*N* = 2), GH15, GH16 (*N* = 3), GH17, GH18 (*N* = 2), GH20, GH26 (*N* = 2), GH27, GH30, GH31 (*N* = 2), GH43 (*N* = 4), GH47, GH55, GH67, GH72 (*N* = 4), GH75, GH95, GH115	[219]
*C. byssicola*	Corn cob (1%), 28 °C, 120 rpm, 7 days	LC–MS/MS	GH2 (*N* = 3), GH3 (*N* = 4), GH5 (*N* = 7), GH6 (*N* = 2), GH7 (*N* = 4), GH10 (*N* = 5), GH11 (*N* = 2), GH12, GH16 (*N* = 2), GH26 (*N* = 2), GH28 (*N* = 3), GH30 (*N* = 2), GH31 (*N* = 2), GH33, GH43 (*N* = 6), GH45, GH47, GH53, GH54 (*N* = 2), GH55, GH62, GH67, GH72 (*N* = 2), GH78, GH93, GH95, GH105, GH115, GH125, GH131	[219]
*C. byssicola*	Banana steam (1%), 28 °C, 120 rpm, 7 days	LC–MS/MS	GH2 (*N* = 3), GH3 (*N* = 2), GH5 (*N* = 7), GH6 (*N* = 2), GH7 (*N* = 4), GH10 (*N* = 5), GH11 (*N* = 2), GH12, GH16 (*N* = 2), GH17, GH26 (*N* = 2), GH28 (*N* = 2), GH30 (*N* = 2), GH31 (*N* = 2), GH33, GH43 (*N* = 5), GH45, GH47, GH53, GH55, GH62, GH67, GH72, GH93 (*N* = 2), GH105,GH115, GH131	[219]
*C. byssicola*	Sugarcane bagasse (1%), 28 °C, 120 rpm, 7 days	LC–MS/MS	GH2 (*N* = 2), GH3 (*N* = 4), GH5 (*N* = 7), GH6 (*N* = 3), GH7 (*N* = 4), GH10 (*N* = 5), GH11 (*N* = 2), GH12, GH17, GH18, GH26 (*N* = 2), GH28 (*N* = 3), GH30 (*N* = 2), GH43 (*N* = 4), GH45, GH53, GH62, GH67, GH72, GH105, GH115, GH131	[219]
*C. byssicola*	Soybean hulls (1%), 28 °C, 120 rpm, 7 days	LC–MS/MS	GH2, GH3 (*N* = 2), GH5 (*N* = 7), GH6, GH7 (*N* = 4), GH10 (*N* = 5), GH11 (*N* = 2), GH17, GH26 (*N* = 2), GH28 (*N* = 3), GH30 (*N* = 2), GH43 (*N* = 2), GH45, GH47, GH53, GH55, GH67, GH72 (*N* = 3), GH131	[219]
*Trichoderma harzianum* EM0925	Corn stover (2%), 30 °C, 3 days	1D-PAGE LC–MS/MS	GH2 (*N* = 5), GH3 (*N* = 6), GH5 (*N* = 3), GH6, GH16 (*N* = 3), GH17, GH18 (*N* = 4), GH20, GH22, GH26, GH7 (*N* = 3), GH28, GH30 (*N* = 8), GH31, GH47, GH54 (*N* = 2), GH55 (*N* = 5), GH61, GH64, GH65, GH71, GH72, GH75 (*N* = 2), GH79 (*N* = 2), GH81, GH92 (*N* = 5), GH125, GH127	[220]
*Trichoderma harzianum* IOC 3844	Partially delignified cellulolignin, 30 °C, 200 rpm, 2 days	1D-PAGE LC–MS/MS	GH2 (*N* = 3), GH3 (*N* = 6), GH5 (*N* = 7), GH6, GH7 (*N* = 2), GH10, GH11 (*N* = 3), GH15 (*N* = 2), GH16 (*N* = 2), GH17 (*N* = 2), GH18 (*N* = 3), GH25, GH27, GH54, GH55 (*N* = 4), GH61 (*N* = 2), GH62 (*N* = 2), GH64 (*N* = 2), GH71 (*N* = 2), GH74, GH81, GH92 (*N* = 3), GH95	[221]
*Trichoderma erinaceum* F3	Steam-explosion treated sugar cane bagasse (1%), 30 °C, 200 rpm, 144 h	LC–MS/MS	GH3 (*N* = 3), GH5 (*N* = 2), GH6, GH7 (*N* = 2), GH10 (*N* = 2), GH11 (*N* = 2), GH12, GH13, GH16 (*N* = 2), GH18, GH54, GH55 (*N* = 2), GH62 (*N* = 2), GH71, GH72 (*N* = 2), GH74, GH92, GH127	[222]
*T. reesei* strain QM9414	Cellulose (1%), 28 °C, 200 rpm, 24 h	DIGE, LC/MS–MS	GH3 (*N* = 6), GH7, GH17 (*N* = 3), GH28, GH30, GH64, GH72 (*N* = 2), GH74 (*N* = 5)	[95]
*Mycothermus thermophilus*	Cellulose, wheat bran and rice straw (3:1:1) (5%), 40 °C, 180 rpm, 10 Day	Q-TOF LC/MS	GH1, GH2 (*N* = 2), GH3 (*N* = 5), GH5, GH6 (*N* = 3), GH7 (*N* = 3), GH10 (*N* = 5), GH11 (*N* = 2), GH12, GH15, GH16 (*N* = 3), GH17, GH18 (*N* = 2), GH20, GH26, GH31, GH37, GH43 (*N* = 6), GH45, GH51, GH55 (*N* = 3), GH62 (*N* = 2), GH67, GH71, GH72 (*N* = 2), GH74, GH94, GH109, GH115, GH131 (*N* = 2)	[223]
*Myceliophthora thermophila*	Barley straw (1%),, 45 °C, 250 rpm, 4 days	LC–MS/MS	GH2 (*N* = 4), GH3 (*N* = 6), GH5 (*N* = 3), GH6 (*N* = 2), GH7 (*N* = 4), GH10 (*N* = 3), GH11 (*N* = 4), GH12, GH13, GH16 (7), GH17 (*N* = 2), GH18 (*N* = 2), GH20, GH24, GH25, GH26, GH27, GH28, GH30 (*N* = 2), GH31 (*N* = 2), GH33, GH37, GH43 (*N* = 8), GH47, GH51, GH53, GH55 (*N* = 4), GH62 (*N* = 2), GH67, GH71, GH72 (*N* = 4), GH75, GH76 (*N* = 2), GH79 (*N* = 2), GH81, GH92, GH93 (*N* = 2), GH95, GH114, GH115 (*N* = 2)	[224]
*Talaromyces emersonii* MTCC387	Cellulose (3%) and wheat grain flour (1.5%), 44° C, 250 rpm, 7 days	LC–MS/MS	GH2, GH3 (*N* = 7), GH5 (*N* = 3), GH6, GH7 (*N* = 2), GH10 (*N* = 2), GH12, GH16 (*N* = 2), GH43, GH45, GH57, GH81, GH127, GH131	[155]
*Podospora anserina*	Avicel cellulose/Sugar beet pulp (1%), 27 °C, 100 rpm, 4 days	1D-PAGE LC–MS/MS	GH2, GH3, GH5, GH6 (*N* = 2), GH7 (*N* = 2), GH10, GH15 (*N* = 2), GH27, GH31, GH37, GH55 (*N* = 2)	[196]
*Laetiporus sulphureus*	CMC (2%), 27 °C, 24 days	1D-PAGE LC–MS/MS	GH2, GH3 (*N* = 2), GH5, GH7, GH10 (*N* = 2), GH15, GH16, GH18, GH27 (*N* = 2), GH31, GH37, GH45, GH55, GH92, GH121	[225]
*Pleurotus ostreatus*	Sugar cane bagasse (4%), 27 °C, 24 Days	1D-PAGE LC–MS/MS	GH3 (*N* = 3), GH5 (*N* = 2), GH6, GH7 (*N* = 2), GH10, GH15, GH27 (*N* = 2), GH47 (*N* = 2), GH51, GH76 (*N* = 2), GH92,	[225]
*Peniophora* sp. CBMAI 1063	Standard medium, 28 °C, 140 rpm, 7 days	1D-PAGE LC–MS/MS	GH10, GH13 (*N* = 3), GH15 (*N* = 3), GH16 (*N* = 2), GH18 (*N* = 3), GH20 (*N* = 2), GH28, GH30 (*N* = 2), GH31 (*N* = 3), GH32, GH43 (*N* = 5), GH51, GH72, GH76 (*N* = 2), GH78, GH88, GH92 (*N* = 3), GH95, GH125, GH128	[178]
*P. ostreatus*	Wheat straw, 28 °C, 21 days	LC–MS/MS	GH1, GH2 (*N* = 2), GH3 (*N* = 4), GH5 (*N* = 3), GH6, GH7 (*N* = 3), GH10, GH12, GH13, GH15, GH16, GH20 (*N* = 2), GH27 (*N* = 2), GH28, GH31, GH35 (*N* = 2), GH37, GH47 (*N* = 3), GH51, GH55, GH72, GH76, GH79, GH88, GH105, GH115 (*N* = 2)	[226]
*P. chrysosporium*	Wheat straw, 28 °C, 21 days	LC–MS/MS	GH2, GH3 (*N* = 5), GH5 (*N* = 7), GH6, GH7 (*N* = 4), GH10 (*N* = 5), GH11, GH12, GH13 (*N* = 2), GH17, GH18 (*N* = 5), GH20, GH27, GH28 (*N* = 3), GH30, GH31, GH35, GH37, GH43 (*N* = 4), GH45, GH47 (*N* = 3), GH53, GH61 (*N* = 2), GH71, GH88, GH155	[226]
*Irpex lacteus*	Corn-step solids growth medium, 28 °C, 21 days	LC–MS/MS	GH2 (*N* = 2), GH3 (*N* = 3), GH5 (*N* = 2), GH7, GH15 (*N* = 2), GH30, GH31 (*N* = 2), GH35 (*N* = 3), GH43, GH92 (*N* = 2), GH105, GH125	[226]
*Irpex lacteus*	Wheat straw, 28 °C, 21 days	LC–MS/MS	GH2, GH3 (*N* = 2), GH5 (*N* = 2), GH6, GH7 (*N* = 3), GH10 (*N* = 2), GH35 (*N* = 2), GH43, GH57, GH61, GH74, GH92	[226]
*D. decipiens* oita	Cellulose (1%), 25 °C, 150 rpm, 3 days	LC–MS/MS	GH2 (*N* = 2), GH3 (*N* = 6), GH5 (*N* = 5), GH6, GH7 (*N* = 4), GH10, GH11 (*N* = 3), GH13, GH15 (*N* = 2), GH16 (*N* = 2), GH17 (*N* = 2), GH18 (*N* = 3), GH28 (*N* = 2), GH29, GH30 (*N* = 2), GH31, GH32, GH37, GH43 (*N* = 6), GH45, GH47, GH51, GH55 (*N* = 3), GH71, GH72 (*N* = 3), GH74, GH76, GH79 (*N* = 2), GH92 (*N* = 2), GH93, GH125	[227]
*Trichoderma guizhouense* NJAU4742	Rice straw (1%), 28 °C, 170 rpm, 6 days	1D-PAGE LC–MS/MS	GH1, GH2, GH3, GH5, GH6, GH7, GH10, GH11, GH12, GH13, GH15, GH16, GH17, GH18, GH20, GH23, GH24, GH25, GH27, GH28, GH30, GH31, GH35, GH39, GH43, GH47, GH50, GH54, GH55, GH61, GH62, GH64, GH65, GH67, GH71, GH72, GH74, GH75, GH76, GH79, GH81, GH92, GH95, GH125, GH127, GH128	[228]
*Postia placenta*	Aspen wafer sections, solid-state fermentation	LC–MS/MS	GH3, GH5 (*N* = 4), GH10, GH16 (*N* = 2), GH27, GH28, GH47, GH51, GH55 (*N* = 3), GH79	[229]
*Gloeophyllum trabeum*	Aspen wafer sections, solid-state fermentation	LC–MS/MS	GH2, GH3 (*N* = 3), GH5 (*N* = 4), GH10 (*N* = 2), GH12, GH16, GH18, GH20 (*N* = 2), GH27, GH28 (*N* = 3), GH31, GH35, GH47, GH51, GH55, GH72, GH79, GH115 (*N* = 2)	[229]
*Trametes versicolor*	Aspen wafer sections, solid-state fermentation	LC–MS/MS	GH2, GH3 (*N* = 4), GH5 (*N* = 3), GH6, GH7 (*N* = 4), GH10 (*N* = 2), GH12 (*N* = 2), GH18, GH20 (*N* = 2), GH27, GH28 (*N* = 3), GH30, GH31, GH35, GH43 (*N* = 2), GH47, GH51, GH53, GH79, GH92 (*N* = 2), GH115, GH131	[229]
*Stereum hirsutum*	Aspen wafer sections, solid-state fermentation	LC–MS/MS	GH3, GH5 (*N* = 3), GH6, GH7, GH10 (*N* = 2), GH15 (*N* = 2), GH18 (*N* = 3), GH28 (*N* = 2), GH72, GH75, GH79	[229]
*P.chrysosporium* RP-78	Avicel cellulose (2%), 37 °C, 150 rpm, 12 days	1D-PAGE LC–MS/MS	GH5 (*N* = 4), GH6, GH7 (*N* = 4), GH10 (*N* = 4), GH11, GH12, GH15, GH18, GH43 (*N* = 3), GH28 (*N* = 2), GH31, GH45, GH55, GH74 (*N* = 2), GH131	[230]
*T. versicolor* BAFC 266	Avicel cellulose (2%), 27 °C, 150 rpm, 12 days	1D-PAGE LC–MS/MS	GH3, GH5 (*N* = 4), GH6, GH7 (*N* = 3), GH10 (*N* = 3), GH12 (*N* = 2), GH18, GH21, GH30, GH31, GH32, GH43, GH45, GH47, GH51, GH53, GH74, GH78, GH92, GH131(*N* = 3)	[230]
*Chrysoporthe cubensis* LPF-1	Wheat bran, semi-solid fermentation, 28 °C, 7 days	1D-PAGE LC–MS/MS	GH1 (*N* = 2), GH2 (*N* = 2), GH3 (*N* = 9), GH5 (*N* = 2), GH6 (*N* = 2), GH7 (*N* = 3), GH10 (*N* = 3), GH11 (*N* = 3), GH12, GH16 (*N* = 3), GH17 (*N* = 3), GH27 (*N* = 3), GH28 (*N* = 4), GH29 (*N* = 2), GH35 (*N* = 2), GH39, GH43 (*N* = 3), GH45 (*N* = 2), GH47 (*N* = 2), GH51, GH54, GH64, GH67, GH71, GH72, GH74, GH78 (*N* = 4), GH79, GH81, GH93 (*N* = 3), GH106 (*N* = 2), GH115, GH128, GH131 (*N* = 2), GH132, GH140	[142]
*Aspergillus clavatus* NRRL1	Sugarcane bagasse (1%), 30 °C, 250 rpm, 5 days	1D-PAGE LC–MS/MS	GH3 (*N* = 4), GH7 (*N* = 2), GH5, GH11, GH17, GH27, GH28 (*N* = 2), GH43, GH62 (*N* = 2)	[231]
*Wolfiporia cocos*	Debarked and wiley-milled aspen (0.5%), 5 °C, 150 rpm, 5 days	1D-PAGE LC–MS/MS	GH3 (*N* = 2), GH5 (*N* = 3), GH10 (*N* = 2), GH15, GH16, GH18 (*N* = 2), GH28, GH51, GH79, GH115 (*N* = 2), GH125	[232]
*Ceriporiopsis subvermispora strain B*	Ball-milled bigtooth aspen (0.5%), 26.5 °C, 150 rpm, 3, 5 and 7 days	LC–MS/MS	GH2 (*N* = 2), GH3 (*N* = 2), GH5 (*N* = 8), GH6, GH7 (*N* = 2), GH10 (*N* = 6), GH12, GH13 (*N* = 2), GH15 (*N* = 2), GH16 (*N* = 6), GH18 (*N* = 4), GH20 (*N* = 2), GH27 (*N* = 2), GH28 (*N* = 2), GH30, GH3 (*N* = 3), GH37, GH43, GH47, GH51, GH55 (*N* = 2), GH72, GH74, GH78, GH79 (*N* = 2), GH88, GH89, GH92 (*N* = 3), GH95, GH115 (*N* = 2)	[233]

In addition to the identification of novel enzymes and regulatory mechanisms, proteomic studies are useful in selecting substrate-specific lignocellulolytic bacterial and fungal strains (Table 1). For example, through a proteomic study, Gong et al., have validated the suitability of *A. niger* ATCC1015 secretome composed of glucanases, esterases, cellulases, mannases, xyloglucanases, xylanases and β-pectinases to breakdown of dicotyledon biomass [63]. While the secretomes of *T. reesei* QM9414, and *Penicillium oxalicum* 114-2 were found to be suitable for the degradation of monocotyledon biomass [57]. Considering the recalcitrance nature of biomass and the abundance of lignocellulolytic enzymes, secretome from different fungi can be combined to improve the biomass degradation. For example, combining *T. reesei* and *A. niger* secretome has been reported to increase the rate of biomass saccharification [58]. Thus, it is important to analyse the secretome of lignocellulolytic strains grown on specific biomass to study the substrate-dependent secretion pattern of GHs, and the identification of novel and powerful GHs for biomass hydrolysis.

### Gel-based and gel-free proteomics approaches

**Fig. 2:**
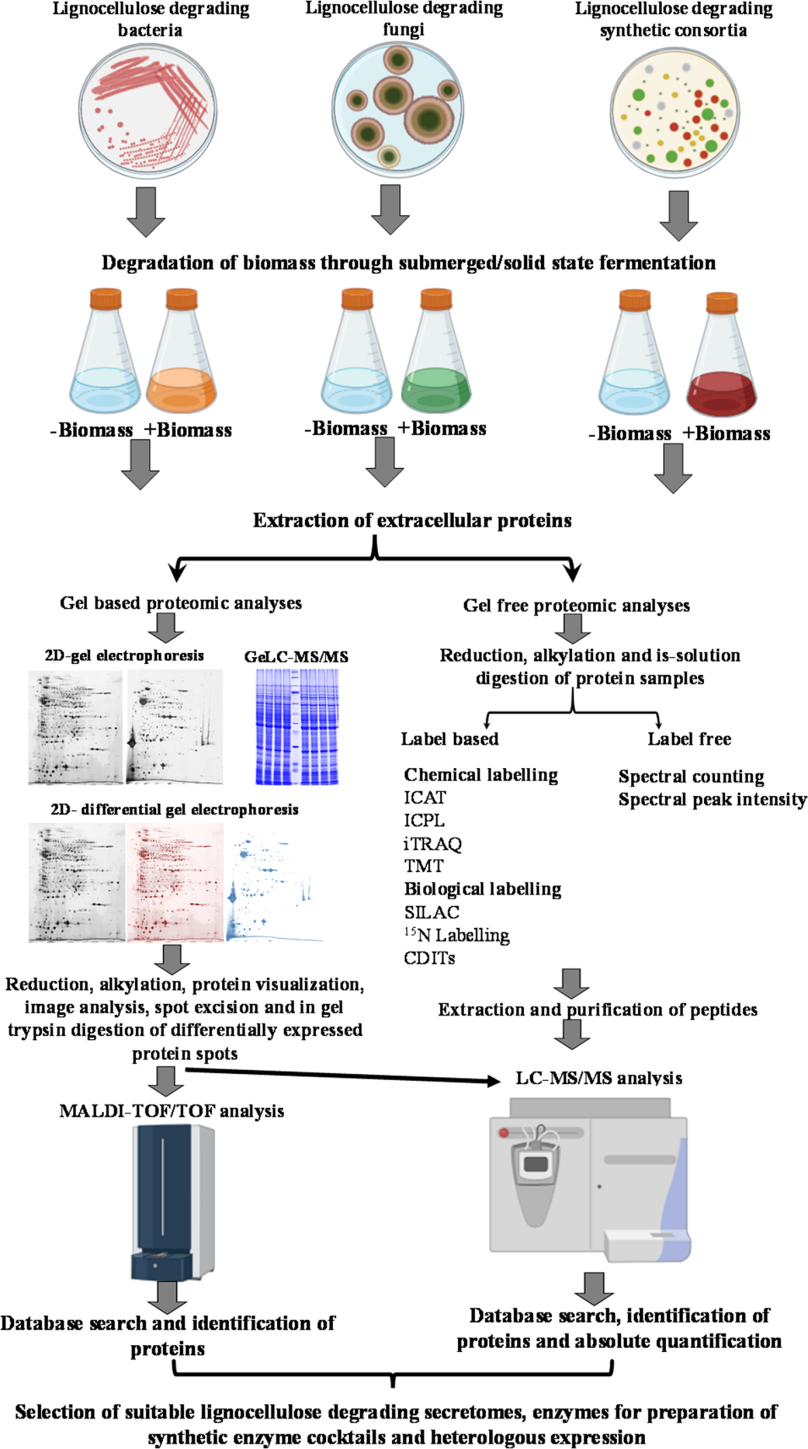
Steps and tools involved in secretomic analysis

As proteins regulate and perform numerous biochemical functions, among the omics approaches, the proteomic approach is very important for accessing the functional status of different life forms. The availability of whole-genome sequences and advances in bioinformatic analysis have made proteomics as one of the best ways to unravel the biological mechanisms and pathways. Furthermore, proteomic analyses are very useful in bridging the knowledge gap between the genetic and functional status of an organism grown under specific conditions [96, 97]. A typical proteomic analysis workflow involves sample preparation, protein separation, mass spectrometric analysis, data acquisition, protein identification and quantification, and the validation of the protein of interest. Sample preparation is an important step in proteomic analysis to obtain accurate and reproducible results. Sample preparation consists on the protein extraction from the samples through physical and enzymatic treatment, solubilization, denaturation, reduction, alkylation, protein labelling, removal of highly abundant proteins to study the low-abundant proteins and tryptic digestion (Fig. 2) [98]. Prefractionation of proteins using SDS-PAGE based on the molecular weight or two-dimensional gel electrophoresis based on isoelectric pH and molecular weight, and prefractionation of tryptic peptides or using reverse-phase high-performance nano liquid chromatography based on the hydrophobicity (Fig. 2). After the separation phase, peptides are analysed by mass spectrometry in which the peptide mixture is ionized and separated to determine the mass-to-charge ratio (*m*/*z*) [99], followed by protein identification using database search tools such as Mascot [100], SEQUEST [101], OLAV [102], MS-FIT (http://prospector.ucsf.edu/), Open mass spectrometry search algorithm (OMSSA) and X! Tandem [103].

In the modern proteomics era, the combination of gel-based and gel-free techniques is successfully used to analyse the complex proteomes using advanced mass spectrometry tools (Fig. 2). Mass spectrometry contains three important components, such as ionization, mass analysis, and detection. Matrix-assisted laser desorption/ionization (MALDI), surface-enhanced laser desorption/ionization (SELDI) liquid chromatography coupled electrospray ionization (ESI) are the major ionization techniques used for peptide analyses [104]. Regarding mass analysers, time-of-flight (TOF and TOF–TOF), ion trap (linear trap, quadrupole and orbitrap trap), Fourier transform ion cyclotron resonance (FT-ICR) and quadrupole-TOF (hybrid analyser) are widely used in proteomic analyses [105, 106]. In recent years, mass spectrometry-based modern proteomics approaches such as top-down [107], bottom-up/shotgun proteomics [108], multidimensional protein identification technology (MudPIT) [109] and gel-enhanced liquid chromatography–mass spectrometry (GeLCMS) [110] have been used to analyse complex proteomes.

The availability of the hybrid mass spectrometer with high sensitivity and mass accuracies such as hybrid linear trap orbitrap mass spectrometer, triple quadrupole mass spectrometer, quadrupole-orbitrap mass spectrometer, triple quadrupole/linear ion trap mass spectrometer, and orbitrap fusion tribrid mass spectrometer, has driven researchers to carry out label -based and label-free relative and absolute quantification of protein expression [111–113]. For label-based mass spectrometric approaches, stable isotope labelling by amino acids in cell culture (SILAC), isotope-coded affinity tag (ICAT), tandem mass tag (TMT) and isobaric tags for relative and absolute quantitation (iTRAQ) are being used to spike the proteins from various biological samples to analyse and quantify the proteins through LC–MS/MS [114].

Label-free mass proteomic analysis is straightforward, less expensive and less time-consuming as compared to other proteomics approaches. This type of proteomic analysis relies on the tryptic digestion of several thousand proteins from the complex biological samples in solution, LC separation of peptides and quantification of proteins based on peak intensity and/or spectral counting [115]. Given that the label-free proteomic approach simultaneously identifies and quantifies thousands of proteins from the biological samples in a single run, thus it is widely used in the field of such as biomedicine, microbiology, bioenergy, biotransformation, etc., to decode several complex biochemical pathways and its regulation, post-translational modifications, the response of an organism to physical, chemical and environmental factors.

Proteomic approaches are successfully used to identify the carbohydrate-active enzymes (CAZymes) from the secretome of biomass deconstructing microbes [116]. Additionally, proteomic analyses provide the quantitative expression of enzymes in response to the available substrates. Secretomic analysis of lignocellulolytic microbes are advantageous in designing synthetic enzyme cocktails and synthetic microbial consortia for the robust and efficient biomass pretreatment and deconstruction. However, the extraction of a whole set of proteins secreted by bacteria/fungi grow in lignocellulosic biomass remains as a difficult task; this is due to difficulties associated with the elimination of contaminants affecting trypsin digestion and the minimization of contaminating cellular proteins, masking the absolute quantification of secreted proteins. The development of activity-based protein profiling methods is necessary to identify substrate-specific and highly active lignocellulose degrading enzymes to facilitate the formation of effective enzyme cocktails.

### Proteomics in profiling of glycoside hydrolases in bacterial and fungal secretomes

Secretome of lignocellulolytic bacteria/fungi composed of proteins anchored on the cell surface, secreted enzymes and proteins involved in biomass hydrolysis. Thus proteomic analysis of the secretome of lignocellulosic biomass-degrading microorganisms helps to formulate enzyme cocktails for better biomass degradation [10, 117–119]. Comparative analysis of the commercially available enzyme cocktails Celluclast 1.5 L and an enzyme cocktail comprising *T. reesei* secretome that comprised cellobiohydrolases, endoglucanase and β-glucosidase on the sugarcane bagasse deconstruction showed higher glucose yield from *T. reesei* secretome treated sugarcane bagasse than Celluclast 1.5 L treated [120]. The use of fungal secretome would be helpful in reducing the cost and increasing lignocellulosic biomass utilization efficiency. Studies have reported increased cellulolytic activity of commercially available enzyme cocktails when completed with fungal secretomes [121].

Especially secretome/enzymes from fungi have received considerable attention due to their wide range of applications in the food, pulp and paper, textile, biofuel and bioenergy industries [122]. In addition, fungal secretomes can be used in combination with lignin-depolymerizing bacteria to prevent the re-polymerization of low molar mass lignin fractions. For example, Salvachúa et al. have reported that the complementation of *Pleurotus eryngii* secretome rich in laccases with *Pseudomonas putida* KT2440 is useful for lignin depolymerization in solid biomass [123].

The secretome of any organism can be predicted through bioinformatics tools using the available whole-genome data [124, 125]. However, experimental identification of enzymes/proteins present in the secretome is more preferential than bioinformatics prediction as it represents the functional status of a genome [126, 127]. Hence, the secretome of industrially important microorganisms has been studied extensively using traditional proteomic approaches and contemporary cutting-edge proteomic approaches (Table 1). Microorganisms can be stimulated to produce the desired composition of enzymatic cocktails by supplementing different sugars in the growth medium [128–130].

### Secretome of industrially important *T. reesei* strains: an arsenal of lignocellulolytic enzymes

T.* reesei* is one of the well-studied lignocellulolytic fungus at the genome, transcriptome and proteome level as it contains robust cellulolytic and hemicellulolytic systems [131]. Several researchers have reported the potential of *T. reesei* hypercellulolytic mutant Rut C30 to degrade lignocellulosic biomass and analysed its secretome using proteomic approaches. When *T. reesei* Rut C30 is cultured on lignocellulosic biomass, it secretes a variety of hydrolytic enzymes targeting cellulose, hemicellulose and lignin [92]. Complementarily, *T. reesei* Rut C30 can be stimulated to secrete the desired enzyme/enzymes by supplementing the appropriate carbon source in the growth medium and hence becoming a promising candidate for economically feasible lignocellulolytic enzymes production [93]. Replacement of transcriptional factors in *T. reesei* Rut C30 with minimal/artificial transcriptional factors [132] and expression of artificial zinc finger proteins [133] are the promising approaches for increasing cellulase secretion. For example, substituting natural transcriptional factors with minimal transcriptional activators such as ACEII and CREI in *T. reesei* Rut C30 increased the cellulase production [134]. Similarly, the introduction of artificial transcriptional factors such as XYR1VP, ACE2VP, and ACE1VP into *T. reesei* Rut C30 has improved the degradation of pretreated corn stover with increased glucose yield [135]. As a result, *T. reesei* is being successfully used for biofuel production at industrial scale.

Proteotranscriptomic analysis of *T. reesei* and genetic engineering has paved a way to understand and overcome factors limiting lignocelluloses degradation. For example, when *T. reesei* has grown on corn steep liquor as a nitrogen source, in the later stages of the fermentation process proteases were found to have a negative effect on the cellulolytic activity [136]. Since *T. reesei* contains nearly 200 protease encoding genes [137], the knocking out and deletion of all the genes encoding proteases become impossible [138], and this can have an adverse effect on strain’s growth. However, Qian et al., have identified the major proteases in the secretome of *T. reesei* grown on corn steep liquor using biochemical assays, LC–MS/MS analysis and successfully constructed a triple protease mutant strain called ΔP70 with reduced protease activity and enhanced the cellulolytic activity on a six-fold basis [136]. Moreover, CRISPR/Cas9 mediated deletion of the cellulase repressor gene (*ACE1*) and secreted protease genes (*SLP1* and *PEP1*), constitutive expression of cellulase master regulator gene (*XYR1*) and heterologous expression of β-glucosidase CEL3A and invertase SUC1 from *Talaromyces emersonii* and *A. niger*, respectively, in *T. reesei* RUT-C30 has remarkably increase the cellulase, hemicellulase, β-glucosidase and xylanase production [135]. These results have further strengthened the prospects for combining genetic engineering and multi-omics technologies to develop of industrially important strains for biomass conversion.

It has been shown that *T. reesei* Rut C30 cultured on spent hydrolysate model medium as a carbon source produces more lignocellulose degrading enzymes such as cellobiohydrolases and endoglucanases compared to control [139]. Genetic engineering of industrially relevant strains for heterologous expression and secretion of desired enzymes has been proved to be one of the best ways to accelerate the utilization of lignocellulosic biomass. Ayrinhac et al., have genetically modified *T. reesei* to secrete a β-glucosidases (evolved through L-Shuffling of putative glycosidase genes from *Chaetomium globosum*, *T. reesei* and *Neurospora crassa*) with 242-fold, increased cellulolytic activity [140]. The recombinant *T. reesei* secretome that comprised the above mentioned β-glucosidases can enable to perform wheat straw saccharification in combination with a fourfold reduced level of commercial cellulase preparation, when compared to the wild-type strain [140]. In a recent investigation, a two-step hydrolyzation of lignocellulosic biomass was conducted to produce glucose. Briefly, in the first step *T. reesei* secretome was used and in the second step the mixture of *Laetisaria arvalis*, *Artolenzites elegans* and *Trametes ljubarskyi* secretomes were deployed for the maximum biomass degradation [11]. Proteomic analysis of the secretome of *Ganoderma lucidum* grown on sugar cane bagasse revealed the presence of lignin-degrading enzymes, cellulases, hemicellulases, glycoside hydrolases, proteases and phosphatases. Complementarily, the authors have also demonstrated the hydrolytic activity of the identified enzymes using biochemical assays [141].

### Secretomic analysis of phytopathogenic fungi for hunting lignocellulolytic enzymes

Since the strategy of using enzyme cocktails has proven to be more effective than the use of single enzymes in lignocelluloses degradation, the identification and characterization of enzymes from bacterial and fungal secretomes would be useful in formulating enzyme cocktails to accelerate lignocellulosic biomass degradation. In addition to the identification of novel enzymes/proteins from lignocellulolytic microbes (Table 1), secretome profiling of phytopathogenic microbes certainly offers the best opportunity to harness very active lignocellulolytic enzymes (Table 1) [142]. For example, LC–MS/MS-based identification of proteins present in the secretome of a phytopathogenic fungus *Ustilago maydis* showed the presence of carbohydrate-active enzymes (CAZymes), hydrolases and oxido-reductases. Similarly, profiling of *Aspergillus niger, Aspergillus nidulans* secretome has demonstrated their ability to produce several CAZymes, oxidoreductases, proteases, lipases and esterases of biotechnological importance [143]. Additionally, the authors have demonstrated a significant increase in the yield of total sugars and glucose, when *T. reesei* CL847 enzymes were supplemented with *U. maydis* secretome for the saccharification of micronised wheat straw [143]. Secretome analysis of *T. reesei* Rut C3 cultured using a mixture of glucose, disaccharide and alkali-pretreated corn stover as inducers revealed the depletion in the production of β-glucosidase. Hence, a β-glucosidase gene from *Aspergillus aculeatus* was heterologously expressed in *T. reesei* Rut C3 under the pdc1 promoter. As a result, β-glucosidase production has increased 71-fold compared to the parent strain. Subsequent application of the secretome for corn stover degradation led to significant increase in ethanol production (54.2 g L^−1^) [144]

### Secretomic analysis of other important lignocellulosic-degrading microbes

Members of the Aspergillus family such as *Aspergillus nidulans*, *Aspergillus oryzae*, *Aspergillus niger* and *Aspergillus fumigatus* are receiving more attention, due to the presence of the arsenal of biomass-deconstruction enzymes. In several studies, secretomes of various *Aspergillus* species have been successfully used to degrade biomass for biofuels production. *A. fumigatus* is known for its thermostable enzymes such as endoglucanases [145, 146], β-xylosidase [147, 148], β-glucosidases [149] and xylanses [150, 151], thus its secretome is considered as one of the promising candidates for the biomass pretreatment in combination with commercially available enzyme cocktails. As a result, secretomes of several *A. fumigatus* strains were studied (Table 1) [152, 153]. For example, the proteomic analysis of the secretome of *A. fumigatus* AF293 cultured on steam-exploded bagasse revealed the presence of endoglucanases, endo-1,4-beta-xylanases, acetyl xylan esterase, feruloyl esterase, feruloyl esterase C, extracellular GDSL-like lipase/acylhydrolase, beta-glucosidase, glycosyl hydrolases, arabinanases, glucanases, endoglucanases, cellobiohydrolases, arabinofuranosidases, 1,4-beta-d-glucancellobiohydrolyase, swollenin and BNR/Asp-box repeat domain protein [154]. These identified enzymes and proteins are associated with the degradation of cellulose, pectin, xylan, arabinoxylan, mannan, galactomannan, glucomannan, β-1,6-glucan and arabinogalactan.

The LC–MS/MS analysis of the secretome of a thermophilic ascomycete *Malbranchea cinnamomea* grown on sorghum straw showed the presence of a wide range of catalytically active and Mn^2+^/Cu^2+^ dependent cellulases (*N* = 8), hemicellulases (*N* = 9), polysaccharide lyases (*N* = 12), proteases (*N* = 9), glyoxal oxidase (*N* = 1) and other enzymes [63]. In a recent study on the secretome of *Talaromyces emersonii* cultured on cellulose and wheat grain flour highlighted the presence of 93, 13, 14 and 8, glycosyl hydrolases (covering 40 different glycosyl hydrolase families), cellulose-binding modules, carbohydrate esterases and auxiliary activity proteins, respectively [155]. However, a genome analysis of *T. emersonii* has speculated the presence of 221 glycosyl hydrolase genes covering 55 different glycosyl hydrolase families, 57 auxiliary activity protein genes and 34 carbohydrate esterase genes [155]. These studies have further confirmed the substrate-dependent secretion of enzymes, and accessory proteins, and highlighted the need of secretome analysis to formulate the suitable and efficient enzyme cocktails to obtain the best results. More interestingly, Claes et al., succeeded in developing a *Saccharomyces cerevisiae* to secrete seven ligninolytic enzymes such as β-glucosidase, β-xylosidase, xylanase, endoglucanase, cellobiohydrolase I, cellobiohydrolase II and acetylxylan esterase from different fungi, and the engineered *S. cerevisiae* was shown to produce bioethanol from various substrates [156]. Co-culturing of lignocellulolytic bacteria and fungi is an emerging area of research in lignocellulosic biomass degradation. Recent investigation on co-cultivation of *Thermomyces lanuginosus* and *Thermobifida fusca* on corn stalks revealed the rapid degradation of xylan by *T. lanuginosus* that enables the breakdown of cellulose by *T. fusca* through secretion of multiple glycoside hydrolases [89]. However, detailed studies on the secretome of co-culturing microbes on different lignocellulosic biomass are required to harness numerous synergistic mechanisms.

### Proteomics in the identification of AALPMOs from the secretome of biomass deconstructing bacteria and fungi

The presence of numerous genes encoding AA9 LPMO in most of the biomass-degrading *Ascomycetes* and *Basidiomycetes*, substrate-dependent expression patterns confirming their role in the deconstruction of biomass along with other major hydrolytic enzymes. Accumulating scientific data highlighting the role of auxiliary proteins in lignocellulose deconstruction, for example, LPMOs [50, 65, 157] enhance the lignocellulose deconstruction through oxidative cleavage, which in turn eases the activity of other major enzymes [65, 157, 158]. AA9 LPMOs disrupt the recalcitrant ultrastructure of cellulose by introducing nicks and weakening its mechanical strength through oxidation, thereby allowing rapid deconstruction of cellulose by cellulases [159]. A recent study has demonstrated that the heterologous expression of a thermostable LPMO of *Talaromyces cellulolyticus* in *T. reesei* significantly enhanced the breakdown of delignified corncob and cellulose [160]. In addition, a LMPO called PoLPMO9A from *Pleurotus ostreatus* has improved the degradation of natural and kraft lignin by a versatile peroxidase through H_2_O_2_ production and introducing structural modifications [161]. In a recent investigation, two LPMOs such as AtAA9A and AtAA9B from *Aspergillus tamarii* were identified to cleave the β-(1 → 4)-glucan linkage in xyloglucan [162]. Previously, it was believed that LPMOs were present in bacteria, fungi and certain viruses, but researchers identified LPMOs from the digestive tract of insects that thrives on lignocellulose biomass. Sabbadin et al., have identified more than 20 AA15 LPMOs accounting for 20.2% of the gut proteome of *Thermobia domestica* (firebrat). Furthermore, in *T. domestica’s* gut several GHs belong to GH1, GH2, GH5, GH9, GH13, GH16, GH18, GH20, GH27, GH30, GH31, GH35, GH38 and GH65 were identified [163]. Similarly, proteomic analysis of the tracheal and mid gut region of *Drosophila melanogaster* showed the presence of two AA15 LPMOs such as DmAA15A and DmAA15B [163]. Further evaluation of these novel LPMOs and their heterologous expression in a suitable lignocellulose degrading fungi/bacteria is expected to built-up a new experimental platform to enhance biomass utilization.

### Substrate-dependent expression of AALPMOs in lignocellulolyitc microbes

Secretome profiling of *Gloeophyllum trabeum* and *Pleurotus ostreatus* cultured on sugarcane bagasse showed the involvement of secreted oxalate decarboxylase, intracellular AA6 quinone oxidoreductases along with cellulases, hemicellulases and auxiliary proteins in deconstructing lignocellulosic biomass [164]. Similarly, proteomic analysis of *T. reesei* Rut C-30 grown on sugar cane bagasse and various cellulosic substrates revealed the secretion of AA9 family enzymes (EGIV, EGVII), whereas AA9 family enzyme ECIV was secreted when *T. reesei* Rut C-30 was grown on xylose [165]. Genomic analysis of ascomycete *Podospora anserina* showed the presence of 39 genes encoding auxiliary activity (AA) enzymes (AA9, *N* = 33; AA11, *N* = 5 and AA13, *N* = 1). However, when it grew on sugar beet pulp, avicel cellulose and soybean hulls, 7, 14 and 20 AA9 enzymes were secreted, respectively [166, 167]. It is worth mentioning that *P. anserina* strain S mat + (CBS143333) harbours 146 genes encoding different AA enzymes (AA1 = 16, AA2 = 4, AA3 = 30, AA4 = 4, 2, AA6 = 1, AA7 = 32, AA8 = 9, AA9 = 33, AA11 = 6, AA12 = 4, AA13 = 1 and AA16 = 1). Nonetheless, CBS143333 secretes 60 AA enzymes (AA1 = 4, AA2 = 1, AA3 = 12, AA5 = 1, AA7 = 15, AA8 = 7, AA9 = 17, AA12 = 2 and AA16 = 1) when cultured in medium containing glucuronoarabinoxylan and wheat straw lignin isolate [168]. Substrate-dependent expression of several cellulases, hemicellulases, ligninolytic enzymes and LPMOs acting on cellulose and hemicellulose by *P. anserina* offers an opportunity for the researchers to prepare custom-designed enzyme cocktails for the maximum utilization of lignocellulose biomass at industrial level [169]. Recently, a novel AA16 from *Aspergillus aculeatus* was identified through proteomic analysis and was expressed in *Pichia pastoris*. Further analysis revealed its oxidative cleavage property on cellulose and showed that increased *T. reesei* cellobiohydrolase I activity synergistically [33]. A recent investigation has showed that LPMO ScAA10C from *Streptomyces coelicolor* photocatalytically induced cellulose oxidation [47, 137]. In addition, AA10 called *Sg*LPMO10A identified from *Streptomyces griseus* was reported to enhance cellulose degradation by GHs [170]. Similarly, the AA10 identified from *Photorhabdus luminescens* contains isoleucine in place of conserved alanine (present in other bacterial LPMOs) in its active site with the ability to act on α‐ and β‐chitin and C1‐oxidation [48]. These findings highlight and reinforce the role of functional proteomics in the identification of appropriate enzyme cocktails with necessary LPMOs for efficient biomass utilization.

### Probing microbial secretomes for lignin-degrading enzymes

Secretomic analysis of *Fusarium solani* MYA 4552 grown on medium containing solid wood rich in lignin revealed the presence of several proteins such as laccases, oxidoreductases, dioxygenase, superoxide dismutases and catalase. Among them, laccases and Mn-independent peroxidase were found to be the major enzymes responsible for the lignin depolymerization [171]. Similarly, proteomic studies have revealed that lignin degradation in *Sphingobacterium* sp. T2 and *Pseudomonas putida* A514 was carried out by Mn dependent superoxide dismutases (Sod1 and Sod2) [172] and Mn independent B subfamily dye-decolorizing peroxidases (DypBs 1 and 2) [173], respectively. Conversely, *Enterobacter lignolyticus* SCF1 depolymerizes lignin using catalases, peroxidases, oxidoreductases, ABC transporter proteins, enzymes involved in the glutathione biosynthesis pathway and the 4-hydroxyphenylacetate pathway [174]. Whereas proteomic investigation of co-cultivation of *Rhodococcus* strains on lignin-containing medium has shown that the depolymerization of lignin through up-regulation of extracellular peroxidases and oxidases, as well as enzymes involved in the β-ketoadipate and the phenylacetic acid pathway for metabolism of lignin degradation products [175].

A recent proteomic investigation on the outer membrane vesicles of *Pseudomonas putida* KT2440 sheds novel insights on the catabolism of lignin. Following important enzymes such as vanillin dehydrogenase, vanillate *O*-demethylase, 4-carboxymuconolactone decarboxylase, 3-oxoadipate enol-lactonase, betaketoadipyl-CoA thiolase, reductases (FMN-dependent NADH-azoreductase and glutathionyl-hydroquinone reductase), alcohol dehydrogenases, ABC transporter substrate-binding proteins (phosphonate, methionine and polyamine) and quercetin-2,3-dioxygenase are selectively packed inside the outer membrane vesicles [176]. Interestingly, supplementation of outer membrane vesicles from wild-type *P. putida* KT2440 cultured in lignin-rich medium enhanced the growth of *P. putida* KT2440 mutant strains lacking protocatechuate 3,4-dioxygenase and 4-hydroxybenzoate 3-monooxygenase [176]. These encouraging findings certainly offer a new avenue to overcome the hindrance of bacterial growth during lignin depolymerization.

Comparative genomic and proteomic analysis of *Pandoraea* sp. ISTKB cultured in the presence of kraft lignin and vanillic acid have revealed the presence of functionally active uncommon aerobic ‘-CoA’-mediated, *ortho* and *meta* ring-breaking aromatic compound degradation pathways and robust oxidoreductases. In addition, *Pandoraea* sp. ISTKB harbours an active polyhydroxyalkanoate synthesis pathway that can utilize lignin and lignin-derived aromatic compounds as carbon source [177]. In a recent secretomic investigation, marine Agaricomycete *Peniophora* sp. CBMAI 1063 has been shown to produce 2 laccases (Pnh_LAC1 [major secretory protein] and Pnh_LAC2) to degrade lignin. Further analysis involving the addition of the Pnh_LAC1-mediator system has shown that effectively removes lignin from sugarcane bagasse and makes the cellulose and hemicellulose more accessible to CAZymes [178]. Similarly, the secretome of *Podospora anserina* cultured on wheat straw lignin resulted in 20 and a threefold increase in the secretion of laccases and H_2_O_2_-producing enzymes, respectively [168]. These findings emphasize the importance of H_2_O_2_ in the deconstruction of lignin by laccases. Although numerous bacteria and fungi have been reported to depolymerize lignin, yet, only a few have undergone in-depth proteomic analyses (Table 2). Hence, it is necessary to analyse the secretome of lignin-degrading microorganisms to decode the underlying mechanism(s). In addition, the global proteomic analysis of lignin-degrading microbes should be carried out to understand the metabolism of lignin derived aromatic compounds, associated bottlenecks and selection of target genes/pathways for genetic manipulation to develop high efficiency lignin-degrading strains as well as to produce the desired end products.

**Table 2:** List of lignin-degrading enzymes identified through secretomic analysis

Microbes	Ligninolytic enzymes	References
*Doratomyces stemonitis* C8	4-*O*-Methyl-glucuronoylmethylesterase, GMC oxidoreductases and catalases	[206]
*A. fumigatus* AF293	Catalase-peroxidase (KatG), cellobiose dehydrogenase (CDH), catalase B (CatB), FAD-oxidase, laccase, and Cu–Zn superoxide dismutase	[154]
*A. fumigatus Z5*	Laccase, Mn-peroxidase and lignin peroxidase	[149]
*Fusarium verticillioides*	Superoxide dismutase	[207]
*Ceriporiopsis subvermispora*	Mn-peroxidases, laccase, aryl alcohol oxidase	[233]
*Ganoderma lucidum*	Mn-peroxidases, laccases, cellobiose dehydrogenase, glutathione reductases and acyl-CoA dehydrogenase-like protein	[141]
*Penicillium chrysogenum* P33	Oxidoreductases	[211]
*Trichoderma reesei*	Laccase, peroxidase, Cytosolic Cu/Zn superoxide dismutase, Mn superoxide dismutase, bifunctional catalase-peroxidas, aldehyde dehydrogenase, aldehyde oxidase, glutathione reductase, oxidoreductases, etc.	[92]
*Phanerochaete chrysosporium* CIRM-BRFM41	Mn-peroxidases, lignin peroxidases	[234]
*Schizophyllum commune* SH12	Glucose oxidases, alcohol oxidases, aryl-alcohol oxidase, glucooligosaccharide oxidase, cellobiose dehydrogenase, 1,4-benzoquinone reductase and ferric reductase	[72]
*Penicillium echinulatum* 2HH	Isoamyl alcohol oxidases, FAD dependent oxidoreductase and 6-hydroxy-d-nicotine oxidase	[62]
*Lentinula edodes*	Laccase 1, laccase 5, laccase 6, laccase 8, laccase 13, glucooligosaccharide oxidases, glucose oxidases, copper radical oxidase, aryl–alcohol oxidases, Mn-peroxidase 2 and pyranose dehydrogenase	[217]
*Pleurotus eryngii, Irpex Lacteus* and *Pleurotus ostreatus*	Laccase, laccase 4, laccase 5, glyoxal oxidases, pepoxidase, Mn peroxidase, phenol oxidase and diphenol oxidase-A2, GMC oxidoreductases and glyoxylatedehydrogenase	[218]
*Phanerochaete chrysosporium*	Glyoxal oxidases, pepoxidase, Mn peroxidase, phenol oxidase and diphenol oxidase-A2, GMC oxidoreductases and glyoxylatedehydrogenase	[218]
*Clonostachys byssicola*	Alcohol oxidase and catalase/peroxidase	[219]
*Aspergillus fumigatus*	Laccase, isoamyl alcohol oxidases, bifunctional catalase-peroxidase, Cu,Zn superoxide dismutase, Mn superoxide dismutases, glutathione reductase, oxidoreductases, alcohol dehydrogenase, aryl–alcohol oxidase, cytochrome c peroxidase and FAD-dependent oxygenase	[153]
*Chrysoporthe cubensis*	Laccases, catalase-peroxidase, choline dehydrogenase, alcohol oxidase, pyranose dehydrogenase, isoamyl alcohol oxidases, GMC oxidoreductase and FAD binding oxidoreductase	[142]
*Pleurotus ostreatus*	Laccase, versatile peroxidase, glyoxal oxidase, alcohol oxidases, GMC oxidoreductase, oxidoreductase-FAD binding domain,	[164]
*Peniophora *sp. CBMAI 1063	Laccases, glyoxal oxidases	[178]
*Podospora anserina*	Laccases, cellobiose dehydrogenase, GMC oxidoreductase, glyoxal oxidase, FAD-linked oxidases and AA7 oxidoreductases	[168]
*Fusarium solani* MYA 4552	Laccase, Nickel superoxide dismutase, catalase, FAD-oxidoreductase, cellobiose dehydrogenase	[185]
*Irpex lacteus*	Dyp peroxidases, Mn peroxidases, glyoxal oxidases and cellobiose dehydrogenases	[226]
*Phanerochaete chrysosporium*	Lignin peroxidases, GMC oxidoreductases, Mn peroxidases, glyoxal oxidases, cellobiose dehydrogenases and pyranose 2-oxidase	[226]
*Pleurotus ostreatus*	Versatile peroxidase, Mn peroxidase, laccases, and glyoxal oxidases	[226]
*Trichoderma guizhouense NJAU4742*	Catalase-peroxidase 2, isoamyl alcohol oxidases, GMC oxidoreductases, multicopper oxidases, Cu/Zn superoxide dismutases and oxidoreductase	[228]
*Trametes versicolor*	Dye-decolorizing peroxidase 1 and 2, laccase 2, 3 and 4, lignin peroxidase1, 2, 6, 9 and 12, Mn peroxidase 1, 2, 3, 4, 5, 6, 9 and 9	[229]
*Stereum hirsutum*	Laccase 5 and Mn peroxidases	[229]
*Bacillus ligniniphilus* L1	Multicopper oxidases, catalase-peroxidase, superoxide dismutase and glutathione transferas, etc.	[97]
*Trametes trogii MT*	Laccase 4, laccase TilA, Mn peroxidase, lignin peroxidase, cellobiose dehydrogenases and pyranose 2-oxidase, phenoloxidase, aryl-alcohol oxidase, etc.	[235]
*Pleurotus ostreatus*	Laccase 2, laccase 6, laccase 9, laccase 10, versatile peroxidase 1 and Mn peroxidase 1	[236]
*Trametes hirsuta* 072	Lignin peroxidase 9, versatile peroxidase2, Mn peroxidase 2, 3, 4, 5, 6 and 7	[237]
*Pseudomonas putida* A514	B subfamily dye-decolorizing peroxidase (*N* = 2)	[173]
*Phanerochaete chrysosporium*	Glyoxal oxidase, aryl alcohol oxidase, alcohol oxidase (*N* = 2), pyranose oxidase, cellobiose dehydrogenase	[69]

### Metaproteomics in lignocellulosic biomass degradation

Metaproteomics is a promising approach to identify the structure, function and dynamics of the complete proteome of the microbial community present in any environmental sample [179]. In addition, it offers an opportunity to analyse the proteome of solitary species within the microbial community. In general, the metaproteomic analysis involved seven major steps namely, sample collection, cell lysis, protein extraction, protein separation, trypsin digestion, peptide fractionation, mass spectrometry analysis followed by database search for further protein identification [179]. Lignocellulosic biomass degradation is carried out by numerous bacteria, actinomycetes and fungi together by complementing each other through secretion of different CAZymes, esterases and oxidoreductases [180]. Insect gut microbiomes are considered as an endless reservoir for the construction of synthetic microbial consortia for biomass utilization and identification of enzymes of industrial importance [181–184]. Hence gut microbiome of insects has been extensively analysed through whole metagenome sequencing to identify novel glycoside hydrolases, lignin-degrading enzymes, transport proteins, etc. [185–187]. In addition, several cellulase and hemicellulose-encoding genes derived from the insect gut microbiomes have been heterologously expressed [184, 188]. However, integrated metagenomic, metatranscriptomic and metaproteomic analyses are required to analyse the metabolic pathways, enzymes involved in biomass degradation and their regulation at the community level [189–192]. Sulfo-NHS-SS-biotin labelling of metasecretome and biomass bound proteome, i.e. meta-surface-proteome of the biomass-degrading microbial community has been shown to increase the depth of identification of enzymes [193, 194].

When compared to other ‘omics’ technologies, metaproteomics provides more valuable precedence for functional analyses. It can also be used as a precision tool to emphasize the investigation of subtle functional genes from large metagenomic data. Metaproteomics has an enormous perspective in the field of biomass utilization, particularly by identifying the functional metasecretome to give a new angle to environmental catalysis**.** In particular, metaproteomics helps select specific enzymes from the synthetic and naturally occurring microbial communities for heterologous expression in industrially demanded strains, making synthetic enzyme cocktails and augmenting the hydrolysis potential of currently available secretome preparations [195]. For example, metasecretomic analysis of a microbial consortium EMSD5 harbouring members of the phyla *Proteobacteria*, *Firmicutes* and *Bacteroidetes* cultured on corn stover showed the presence of various glycosyl hydrolases especially multiple xylanases followed by cellulases. By breaking down xylan, EMSD5 secretome significantly increased the hydrolysis of pretreated corn stover by commercially available *T. reesei* secretome preparation [196]. Researchers have identified several CAZymes from the metasecretome of switchgrass thermophilic bacterial consortium and rice straw adapted microbial consortia [197, 198].

Though metaproteomic analysis is considered as a promising tool to evaluate the functional state of the microbiota present in the different environmental samples, proteomic information related to the lignocellulosic biomass-degrading microbial community remains limited. Due to challenges associated with metaproteomic analysis such as complexity of the environmental samples, quality of extracted proteins, peptide extraction, requirement of long nano-LC run time, lack of protein sequence database for protein identification, less coverage of proteomic information from low abundant species, hence limiting the application of metaproteomic analysis [198]. In spite of the above mentioned challenges, the development of specific metaproteomic data analysis tools such as MetaProteomAnalyzer (MPA portable) [199], Unipept 4.0 [200], Calis-p [201], ComPIL 2.0 [202], Unipept Desktop [203] and metaQuantome [204] are encouraging the in-depth metaproteomic research. Furthermore, a newly developed mass spectrometer called Trapped Ion Mobility Mass Spectrometer (TIMS) can achieve the acquisition of more than 60,0000 spectra in 2 h of liquid chromatography runs [205]. In light of the newly developed analysis methods, research combining metagenomic and metasecretomic analyses is expected to shed more light on the identification of novel enzymes, synergistic mechanisms and microbial consortia for biomass utilization.

## Conclusion and future perspectives

The replacement of fossil fuels with biofuels produced from renewable sources such as lignocellulosic biomass is an inevitable phenomenon in the coming years due to the necessity for greenhouse gas emissions reduction and the energy crisis. Forestry and agricultural biomass wastes are an abundant and renewable source of carbon for the production of bio-energy and numerous high-value chemicals. At industrial scale, physical, chemical and thermochemical methods are preferably used rather than biochemical methods for biomass utilization. However, a full utilization of biomass is not yet reached, due its chemical recalcitrance.

In addition, reducing the cost of converting biomass into fermentable sugars is a major challenge for biomass-processing industries. Microbes are the major players involved in biomass degradation, nutrient cycling and their application is considered as an environmentally friendly and a promising economically feasible way to obtain fermentable sugars. In recent years proteomic analysis has been used extensively to explore the secretomes of biomass-degrading bacterial/fungal strains and microbial consortia to identify novel enzymes, to decode the substrate-dependent expression of lignocellulases and post-translational modifications.

With the availability of optimized protein extraction protocols, and absolute quantitative proteomic approaches such as ITRAQ and TMT, each protein can be identified and quantified simultaneously in multiple samples. This milestone can enable researchers to design specific enzyme cocktails with the perfect combination and absolute composition of enzymes for an effective biomass pretreatment and utilization. Although currently available proteomic analysis methods are very good for analysing the secretome of microorganisms grown on different biomass, the extraction and purification of secreted proteins from the environmental samples still requires further improvement. Furthermore, several key enzymes identified from the secretome of lignocellulolytic microbes should be heterologously expressed and studied further to validate their involvement in biomass deconstruction.

Lignin provides recalcitrance to lignocellulosic biomass and acts as a barrier against the saccharification of cellulose. Thus, delignification of lignocellulosic biomass is required to achieve a maximum saccharification of cellulose. For a biological deconstruction of lignocellulosic biomass, enzyme cocktails should be complemented by ligninolytic enzymes. Several proteomics studies showed the consecutive secretion of CAZymes (GHs, GTs, PLs, CEs and AAs) in lignocellulolytic bacteria and fungi. Delignification is essential for an effective saccharification and reduction of cellulolytic enzyme consumption. Although enzyme cocktails and microbial consortia have been used for lignocellulosic biomass deconstruction, less attention has been paid to ligninolytic enzymes. Given the advantages of delignification, the engineering of hyper ligninolytic enzymes secreting strains and the development of ligninolytic microbial consortia should be carried out for biomass pretreatment.

In order to develop a lignocellulosic biomass-specific enzyme cocktails, a comprehensive screening of so far identified lignocellulolytic enzymes from the secretomes of various bacteria, fungi and synthetic/natural microbial communities should be done to select potential candidate enzymes, capable of performing at a wide pH and temperature range. Post-translational modifications (PTMs) (for example N- and O-glycosylation) play a key role in the stability and activity of biomass-hydrolyzing enzymes, but most of the secretomic studies lack information about PTMs. Therefore, future studies involving the identification of important PTMs and finding suitable hosts for heterologous production of target enzymes with appropriate PTMs is expected to overcome the low lignocellulolytic efficiency of heterologously produced enzymes.

In addition, the engineering of industrial strains to simultaneously secrete multiple ligninolytic enzymes is required to reduce biofuels and other commercially important chemicals production costs. The combination of the secretome of different organisms is one of the ways to cut down the enzyme production costs and make use of the synergistic effect among the enzymes in biomass hydrolysis. For example, *T. reesei* secretome is rich in endoglucanases and exoglucanases, whereas members of *Aspergillus* secrete more β-glucosidases and combining the secretomes of *T. reesei* and *Aspergillus*, along with a bacterial/fungal secretome-rich in the auxiliary enzymes could improve the biomass hydrolysis and reduce the enzymes’s production costs. Similarly, further attention should be paid on the development of more hyper lignocellulolytic enzymes-secreting strains. The development of enzymes can withstand under harsh reaction conditions through enzyme engineering, development of simple methods for reusing/immobilizing enzyme cocktails to obtain complete biomass hydrolysis.

## Data Availability

Not applicable.

## Abbreviations

MnP: Manganese peroxidase
LiP: Lignin peroxidase
VP: Versatile peroxidase
DyP: Dye-decolorizing peroxidase
SMF: Submerged fermentation
SSF: Solid-state fermentation
SQF: Sequential fermentation
CCP: Commercial cellulase preparations
MALDI-TOF: Matrix-assisted laser desorption/ionization
SDS-PAGE: Sodium dodecyl sulphate–polyacrylamide gel electrophoresis
LC–MS: Liquid chromatography–mass spectrometry
iTRAQ: Isobaric tags for relative and absolute quantitation
CDH: Cellobiose dehydrogenase
GHs: Glycosyl hydrolases
LPMOs: Lytic polysaccharide monooxygenases
AA: Auxiliary activity
PLs: Polysaccharide lyases
CEs: Carbohydrate esterases
GTs: Glycosyltransferases
CAZymes: Carbohydrate-active enzymes
CMC: Carboxy methyl cellulose
MCOs: Mulita-copper oxidoreductases
SILAC: Stable isotope labelling by amino acids in cell culture
ICAT: Isotope-coded affinity tag
TMT: Tandem mass tag
GeLCMS: Gel-enhanced liquid chromatography–mass spectrometry
MudPIT: Multidimensional protein identification technology
FT-ICR: Fourier transform ion cyclotron resonance
PTMs: Post-translational modifications
